# The Effect of Indoor Residual Spraying on the Prevalence of Malaria Parasite Infection, Clinical Malaria and Anemia in an Area of Perennial Transmission and Moderate Coverage of Insecticide Treated Nets in Western Kenya

**DOI:** 10.1371/journal.pone.0145282

**Published:** 2016-01-05

**Authors:** John E. Gimnig, Peter Otieno, Vincent Were, Doris Marwanga, Daisy Abong’o, Ryan Wiegand, John Williamson, Adam Wolkon, Ying Zhou, M. Nabie Bayoh, Neil F. Lobo, Kayla Laserson, Simon Kariuki, Mary J. Hamel

**Affiliations:** 1 Division of Parasitic Diseases and Malaria, Center for Global Health, Centers for Disease Control and Prevention, Atlanta, Georgia, United States of America; 2 Centre for Global Health Research, Kenya Medical Research Institute, Kisumu, Kenya; 3 Eck Institute for Global Health, Department of Biological Sciences, University of Notre Dame, Notre Dame, Indiana, United States of America; Université Pierre et Marie Curie, FRANCE

## Abstract

**Background:**

Insecticide treated nets (ITNs) and indoor residual spraying (IRS) have been scaled up for malaria prevention in sub-Saharan Africa. However, there are few studies on the benefit of implementing IRS in areas with moderate to high coverage of ITNs. We evaluated the impact of an IRS program on malaria related outcomes in western Kenya, an area of intense perennial malaria transmission and moderate ITN coverage (55–65% use of any net the previous night).

**Methods:**

The Kenya Division of Malaria Control, with support from the US President’s Malaria Initiative, conducted IRS in one lowland endemic district with moderate coverage of ITNs. Surveys were conducted in the IRS district and a neighboring district before IRS, after one round of IRS in July-Sept 2008 and after a second round of IRS in April-May 2009. IRS was conducted with pyrethroid insecticides. At each survey, 30 clusters were selected for sampling and within each cluster, 12 compounds were randomly selected. The primary outcomes measured in all residents of selected compounds included malaria parasitemia, clinical malaria (*P*. *falciparum* infection plus history of fever) and anemia (Hb<8) of all residents in randomly selected compounds. At each survey round, individuals from the IRS district were matched to those from the non-IRS district using propensity scores and multivariate logistic regression models were constructed based on the matched dataset.

**Results:**

At baseline and after one round of IRS, there were no differences between the two districts in the prevalence of malaria parasitemia, clinical malaria or anemia. After two rounds of IRS, the prevalence of malaria parasitemia was 6.4% in the IRS district compared to 16.7% in the comparison district (OR = 0.36, 95% CI = 0.22–0.59, p<0.001). The prevalence of clinical malaria was also lower in the IRS district (1.8% vs. 4.9%, OR = 0.37, 95% CI = 0.20–0.68, p = 0.001). The prevalence of anemia was lower in the IRS district but only in children under 5 years of age (2.8% vs. 9.3%, OR = 0.30, 95% CI = 0.13–0.71, p = 0.006). Multivariate models incorporating both IRS and ITNs indicated that both had an impact on malaria parasitemia and clinical malaria but the independent effect of ITNs was reduced in the district that had received two rounds of IRS. There was no statistically significant independent effect of ITNs on the prevalence of anemia in any age group.

**Conclusions:**

Both IRS and ITNs are effective tools for reducing malaria burden and when implemented in an area of moderate to high transmission with moderate ITN coverage, there may be an added benefit of IRS. The value of adding ITNs to IRS is less clear as their benefits may be masked by IRS. Additional monitoring of malaria control programs that implement ITNs and IRS concurrently is encouraged to better understand how to maximize the benefits of both interventions, particularly in the context of increasing pyrethroid resistance.

## Introduction

Insecticide treated nets (ITNs) and indoor residual spraying (IRS) are two of the most effective malaria prevention strategies recommended for use in sub-Saharan Africa. The efficacy of ITNs in reducing malaria prevalence, severe disease and child mortality was first demonstrated in large scale, cluster randomized, controlled trials in 4 sites in Africa [1]. Though formal randomized trials of IRS are less common [2], the historical evidence from malaria control programs is no less impressive [3]. IRS was the main strategy of the Global Malaria Eradication Campaign which resulted in the elimination of malaria from many countries and greatly reduced its burden in others [4]. However, IRS has traditionally been targeted to areas with low and/or seasonal transmission and its recent expansion into high transmission areas has been questioned due to concerns about long-term sustainability [5].

Both ITNs and targeted IRS were being scaled up throughout sub-Saharan Africa during the last 10 years. By 2011, household ownership of ITNs reached 53% while 11% of the at risk population in the African region was protected by IRS [6]. During the past decade, as malaria control interventions have been scaled up in Africa, substantial reductions in malaria illness have been observed and malaria specific mortality declined by 42% by 2013 [7].

Recent studies and reviews of IRS and ITNs focused on direct comparisons of the efficacy and cost-effectiveness of these interventions. Several studies concluded that both were equally efficacious but that the cost-effectiveness of each intervention was dependent upon the unique setting in which it was implemented [8–11]. With increasing funding for malaria prevention in Africa, a small number of countries have been able to implement both ITNs and IRS although no country has achieved universal coverage with either intervention. As programs expanded, IRS has been implemented in areas where there was already high coverage of ITNs and ITNs were being distributed in areas that were already covered under IRS programs. However, while support for malaria prevention has increased substantially in the last decade, it is not unlimited and has stagnated since 2009. The implementation of both ITNs and IRS in the same location at the same time raises questions about whether this is the most efficient use of finite resources. If the implementation of both provides substantial added benefit over either alone, then malaria control programs may be justified to implement each in the same areas. However, it is possible that ITNs and IRS are both targeting the same population of mosquitoes—those that enter houses to feed on the human inhabitants—and therefore will provide limited added benefit when implemented concurrently. It is also possible that even where an additional benefit may be observed, the high cost of implementing both interventions means this approach may not be cost-effective.

Several non-randomized studies have assessed the combined impact of ITNs and IRS. Observational studies in Equatorial Guinea and Mozambique indicated that the effects of both ITNs and IRS can be additive and recommended scaling up both ITNs and IRS where possible [12]. Similarly, a multi-country study reported added benefits of combining ITNs and IRS, particularly in areas of moderate to high transmission [13]. However, studies in Eritrea [14] and Burundi [15] found no evidence for an added effect of combining these vector control tools. The three cluster randomized studies on the combined efficacy of ITNs and IRS also had inconsistent results: in Tanzania, the addition of IRS to ITNs contributed significant added protection compared to ITNs alone [16, 17] whereas in Benin and the Gambia, no added benefit was observed [18, 19]. Additional studies in other ecological and epidemiological regions are necessary to assess the effectiveness of combining IRS and ITNs to ensure the efficient use of scarce resources for malaria control and prevention.

Kenya provided an opportunity to observe the efficacy of ITNs and IRS either alone or in combination in a programmatic setting in an area of moderate to high transmission. Through heavily subsidized distributions at government health facilities since 2004 and a national mass campaign in 2006, household coverage of ITNs in Kenya reached 50.7% [20]. In 2008, with funding from the US President’s Malaria Initiative, Kenya implemented IRS in the former Rachuonyo District, a setting with perennial malaria transmission located along Lake Victoria. The initial round of IRS spraying was conducted between July and September of 2008 and a second round was conducted in April 2009. In a previously reported study, conducted in the same district and a comparison district that did not receive IRS, the combination of IRS and ITNs was shown to significantly reduce the incidence of malaria infection, by 62%, when compared with ITNs alone in a cohort of persons cleared of infection at study onset [21]. In this paper, we describe the results from three cross-sectional household surveys conducted in Rachuonyo District and a neighboring unsprayed comparison district to assess the combined effect of implementing ITNs and IRS in western Kenya.

## Materials and Methods

### Study Design

This study was an observational study of the impact of ITNs and IRS in western Kenya. ITNs were available through several distribution channels while IRS was conducted in Rachuonyo district by the Kenya Division of Malaria Control with support from the US President’s Malaria Initiative as part of their malaria control program. Three cross-sectional surveys were conducted to assess the impact of these programmatic interventions on malaria related outcomes.

### Study Population

**Fig 1:**
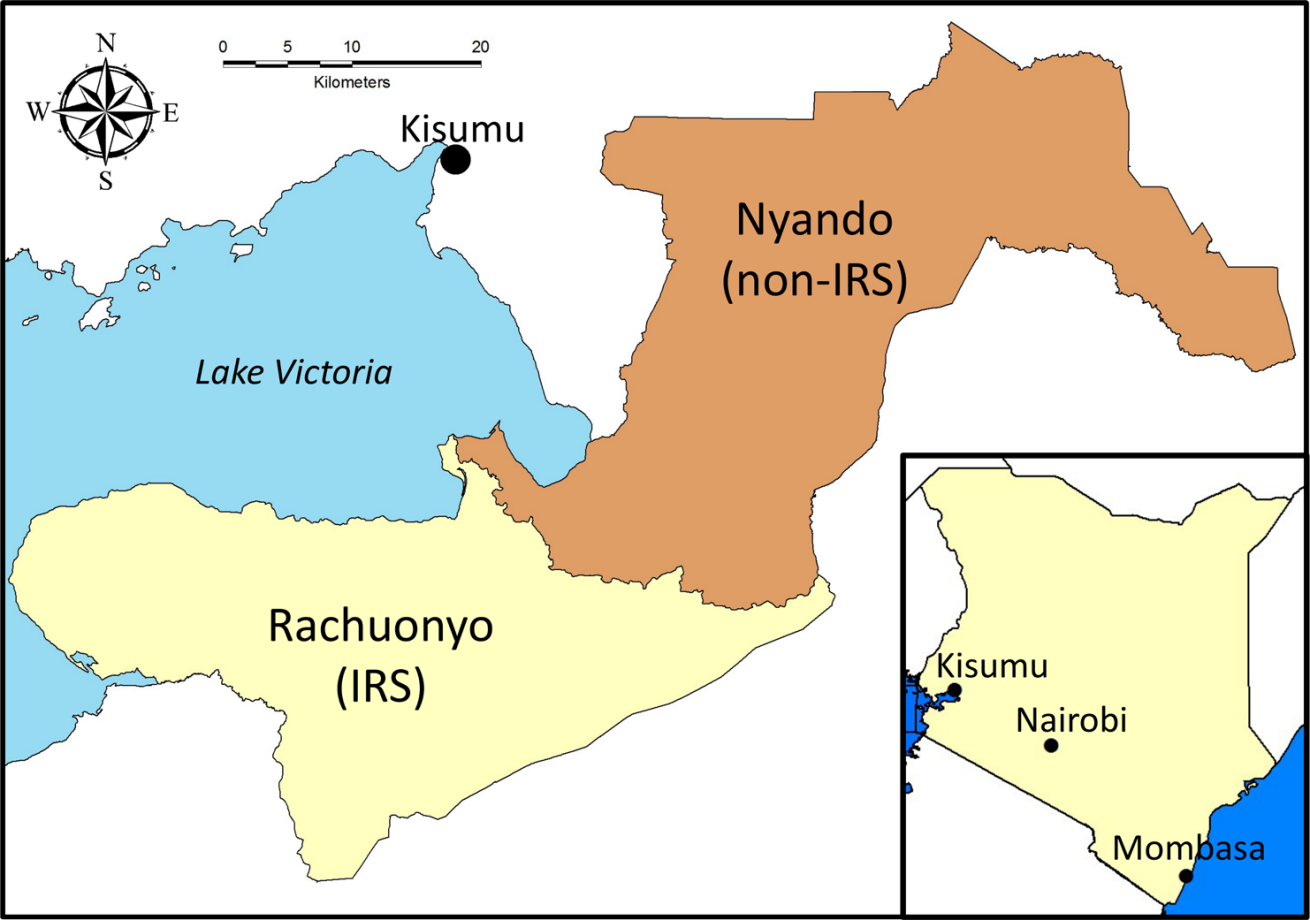
Map of Rachuonyo and Nyando Districts in western Kenya. Rachuonyo district received one round of IRS in July-September 2008 and a second round in May-April 2009.

**Fig 2:**
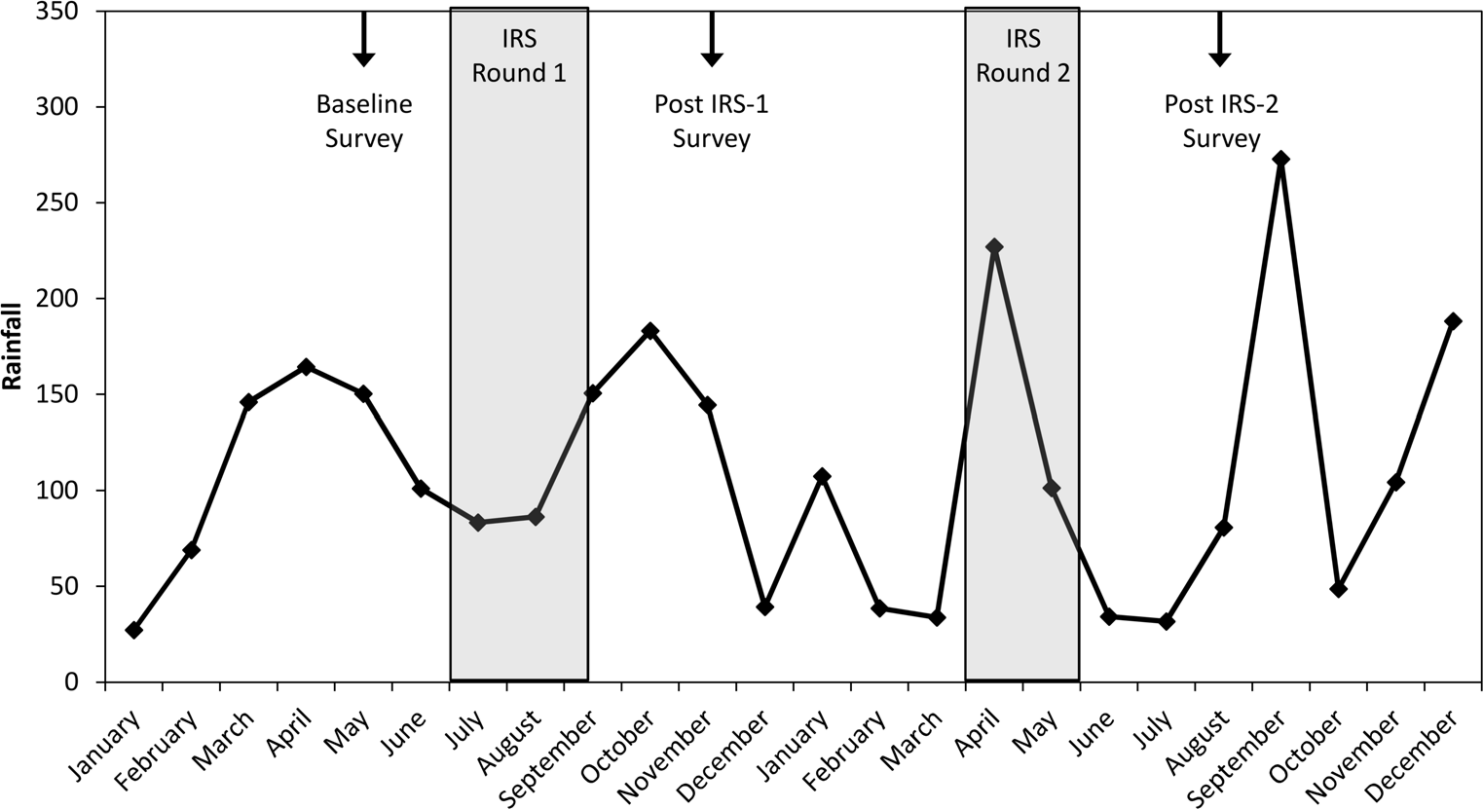
Total monthly rainfall at Kisumu airport from January 2008 through December 2009. The timing of the IRS campaigns are indicated by the shaded areas while arrows indicate the timing of the three cross-sectional surveys.

This study was conducted through an ongoing collaboration between KEMRI and CDC. The study was conducted in two former districts in what was Nyanza Province, Western Kenya (Fig 1). Rachuonyo District (referred to hereafter as the IRS district) was located along the southeastern edge of the Winam Gulf of Lake Victoria. Nyando District (referred to hereafter as the non-IRS district) was located along the eastern edge of the Winam Gulf and northeast of Rachuonyo District. Both districts lie at approximately 1100 m elevation along the lakeshore but rise to over 1500 m away from the lake. The residents of both districts were primarily of the Luo ethnic group. Additionally, members of the Kalinjin ethnic group primarily resided in the highland areas of both districts and large numbers of people from the Kisii ethnic group lived in southern Rachuonyo District. Residents generally lived on compounds that included several structures and that housed extended families. Rainfall at the Kisumu airport, located approximately 40 km from the Nyando/Rachuonyo border averages approximately 1200 mm per year. Rainfall and malaria transmission follows two annual peaks. The long rains occur from March to June and the short rains generally occur in October to November. However, rainfall and malaria transmission occur year round in lowland areas of both districts. Transmission at higher elevations in western Kenya is lower and more seasonal. *Anopheles gambiae*, *Anopheles arabiensis* and *Anopheles funestus* are common throughout western Kenya but *An*. *arabiensis* is the dominant species observed inside houses in these districts (N. Bayoh, unpublished data). Total monthly rainfall from January 2008 to December 2009 is shown in Fig 2 with the timing of the IRS campaigns and the cross-sectional surveys.

### Interventions

The Kenya Division of Malaria Control began distributions of ITNs in 2004 at a subsidized price for pregnant women and children <5 years. Beginning in 2007, nets were distributed free of charge to pregnant women and children <1 year. The routine distribution system was supplemented by social marketing of subsidized nets and a mass campaign in 2006 targeting all children <5 years in endemic regions. Most of the nets distributed were either PermaNets (Vestergaard-Frandsen) or Olyset nets (Sumitomo Chemical Co.). Indoor residual spraying was originally targeted for epidemic-prone districts in the highlands of western Kenya. Beginning in 2008, a single lowland endemic district (Rachuonyo) was also targeted for IRS. The first round was conducted in July-September 2008 with lambda-cyhalothrin (ICON CS, Syngenta) and a second round was conducted in April-May 2009 using alphacypermethrin (Fendona, BASF).

### Cross-sectional surveys

Three cross-sectional cluster sample household surveys were conducted in the study districts. A baseline survey (baseline survey) was conducted in May, 2008, in the low malaria transmission season and before IRS; the first post-IRS survey (post IRS-1 survey) was conducted in November, 2008, in the low malaria transmission season, and 3 months after the first round of IRS; and the second post-IRS survey (post IRS-2 survey) was conducted in August, 2009, just after the end of the main malaria transmission season and 4 months after the second round of IRS.

Prior to each survey, thirty enumeration areas defined by the Kenya Census Bureau were randomly selected in each district with the probability of sampling proportional to the population size of the enumeration area at the last census (1999). A team was sent to obtain GPS coordinates of each compound within the selected enumeration areas. The compounds were mapped using PDAs and GPS Sample software [22]. The software also generated a random sample of 12 compounds per enumeration area and assisted in navigation back to the selected compounds.

Six teams composed of an interviewer and a sample taker were employed in each district. Teams visited one enumeration area each day and visited all of the selected compounds. Within each selected compound, all compound members ≥6 months of age were eligible to participate in the survey. Using a standardized questionnaire, participants who provided informed consent were interviewed, or for those 12 years of age or younger, the primary caretaker was interviewed. The interviewer recorded the participant’s age, gender, recent use of anti-malarials and whether he/she slept under a net the previous night. The interviewers also recorded housing characteristics, specifically the type of roof, the type of walls and whether the structure had been sprayed with insecticide in the last 12 months. All nets in the household were examined and the interviewer recorded the brand of net, its age and, if the net was not a long-lasting net, whether it was treated with insecticide in the last 12 months. The pre-IRS survey was collected on paper forms while data for the other two surveys were recorded on PDAs in forms developed in Visual CE (Syware Inc., Cambridge, MA).

A fingerstick sample of peripheral blood was taken from each participant and was tested for malaria using a rapid diagnostic test (RDT; Paracheck, Orchid Biomedical Systems, Goa, India) to guide clinical care, anemia using a Hemocue machine (HemoCue AB, Ǻngelholm, Sweden), and a blood smear was made for diagnosis of malaria and parasite density determination by microscopy. A filter paper sample of blood was also taken for serological and/or molecular tests of malaria infection.

### Statistical analysis

Univariate analyses comparing characteristics between each study district were done for each survey by logistic regression for binary variables, by χ^2^ for categorical variables and by linear regression for continuous variables. All models adjusted for clustering in the analyses. The prevalence of parasitemia, clinical malaria and anemia among participants living in the IRS district were compared with that of participants living in the non-IRS district using logistic regression. Initial analyses were done with district, survey and an interaction between district and survey as predictor variables. Because the district by survey interaction terms were significant, further analyses were done separately for each survey. Initial analyses also compared the effect of ITNs, untreated nets or no nets on the prevalence of *P*. *falciparum* parasitemia. Because there was no significant difference in parasite prevalence between users of ITNs and untreated nets, the two were combined in all subsequent analyses. Similarly, because some houses within the IRS district did not receive IRS, the prevalence of *P*. *falciparum* parasitemia among persons living in houses that were sprayed or unsprayed was compared among persons living in the IRS district. No differences in parasite prevalence were observed and district was used as a proxy for IRS in subsequent analyses unless otherwise stated. Clinical malaria was defined as current temperature ≥ 37.5° centigrade, or reported fever in the prior 24 hours, plus positive malaria parasitemia by blood smear. Anemia was defined as Hb<8gm/dl. Persons living at or above 1500 m were considered to be in a highland area where mean temperatures are at the edge of suitability for malaria transmission.

For the initial analyses, vector control was categorized as 1) both ITNs and IRS, 2) ITNs only, 3) IRS only or 4) neither. In these models, IRS was measured directly and district was not used as a proxy for IRS. To estimate the added benefit of ITNs in a district that had been sprayed, additional models were tested using an interaction term between ITNs and IRS. In these models, district was used as a proxy for IRS. To reduce the bias due to confounding variables, participants from the IRS district and the non-IRS district were matched at each survey using propensity score matching [23, 24]. Briefly, logistic regression was run using district as the outcome variable and all potential confounders for the association between district and the outcomes included as predictors such as use of an ITN the previous night, ITN coverage in the cluster, elevation, gender, age, history of ACT use, reported use of mosquito coils or other household insecticides, recent history of travel, house construction, and the presence of open eaves. The resulting propensity scores of being in the IRS district were used to match individuals from the non-IRS district to individuals from the IRS district using 1:1 nearest neighbor matching with a maximum probability difference of 0.1. Unmatched subjects were not included in the final models; however, all data were used in estimates of descriptive characteristics.

For each analysis, the models controlled for gender, age (categorized as <5 years, 5 to 14 years and ≥15 years), elevation (<1500m versus ≥1500m), house type (traditional, semi-permanent, permanent), the use of coils or other household insecticides and reported history of antimalarials in the previous two weeks.

All analyses were done in SAS version 9.3 (SAS Institute, Inc., Cary, NC). Descriptive characteristics were estimated using the SURVEYFREQ procedure for categorical variables and the SURVEYREG procedure for continuous variables to estimate confidence intervals adjusted for repeated measures within the same cluster. Logistic regression models adjusting for the effect of cluster were used to compare binomial characteristics between the districts. Chi square analysis was done for categorical variables and general linear models were used for continuous variables. Multivariate logistic regression models of the prevalence of parasitemia, clinical malaria and anemia were developed using the GENMOD procedure. Age, gender, history of ACT use, use of mosquito coils are household insecticides, elevation and house type were included potential modifiers. All models were adjusted for repeated measures within the same cluster using generalized estimating equations and assuming the independence correlation structure [25]. All models assumed 5% as the level of significance.

### Ethics statement

The protocol for this study was reviewed and approved by the Ethics Review Committee of the Kenya Medical Research Institute. The protocol was also reviewed and determined to be program evaluation by the Centers for Disease Control and Prevention. Written informed consent was obtained from all participants or, in the case of minors, from their guardians. For older children (aged 8 to 17), assent was obtained in addition to the consent of the guardian.

## Results

### Population characteristics

Baseline characteristics of the population are shown in Table 1 along with univariate analyses comparing between the two districts. Residents in the IRS district on average lived at higher elevations (1,406 m vs 1,256 m in the IRS district and the non-IRS district, p<0.001) although there was no difference in the proportion living above 1500 m. Persons living in the IRS district were more likely to live in a semi-permanent house structure compared with the non-IRS district (69.6% vs 52.3%, p<0.001) but were less likely to have houses with the eaves closed (10.0% vs 27.8%, p<0.001). Before the implementation of IRS in Rachuonyo, persons in the non-IRS district were more likely to live in a house that had received IRS although only 1.3% of house owners in the non-IRS district reported their houses being sprayed in the previous 12 months compared with 0.0% in IRS district. Overall, residents of the non-IRS district were more likely than those in the IRS district to have slept under a net the previous night (64.7% vs 52.9%, p = 0.008) although there were no differences among children <5 years of age or among persons ≥15 years of age. However, use of an ITN was higher in the non-IRS district overall, (45.3% vs. 34.0%, p = 0.001), and the differences were observed in all age groups. Persons in the non-IRS district were more likely than those in the IRS district to report a recent history of fever (32.3% vs 26.0%, p = 0.035). Differences in reported history of fever were observed in adults aged ≥15 years of age (38.6% vs. 31.5%, p = 0.043) but not among other age groups. There were no differences in reported history of anti-malarials or ACT use among any age group at the baseline survey with the exception of anti-malaria use among persons ≥15 years of age which was higher in the IRS district (13.9% vs. 9.1%, p = 0.031).

**Table 1:** Characteristics of the survey population during the baseline survey (May 2008). Values are presented with 95% CI and sample size in parentheses. P-values represent univariate comparisons between IRS and non-IRS districts. Comparisons that were statistically significant at p<0.05 are indicated in bold. All comparisons controlled for clustering within enumeration areas.

	Non-IRS District	IRS District	P value
N	1016	1423	
Median age in years (IQR)	15.5 (6–32)	14 (6–30)	**0.029**
Female	56.8 (54.0–59.6, 1016)	57.5 (55.0–59.9, 1423)	0.735
Caretaker with some secondary) education (for children <5 years	22.9 (15.0–30.8, 175)	20.9 (14.9–26.9, 254)	0.906
Living with biological mother (children <5 only)	95.0 (91.6–98.4, 180)	95.8 (93.2–98.5, 265)	0.709
Median elevation (m) (IQR)	1,256 (1,181–1,352)	1,406 (1,190–1,471)	**<0.001**
Elevation ≥1500 m	7.9 (3.7–12.0, 1016)	13.8 (9.3–18.4, 1423)	0.397
House type^1^			
Traditional mud hut	20.8 (15.8–25.8, 204)	15.5 (11.3–19.8, 211)	
Semi-permanent (corrugated iron roof)	52.3 (46.2–58.5, 513)	69.6 (64.3–74.9, 946)	
Permanent (concrete or stone walls)	26.8 (20.9–32.8, 263)	14.9 (10.6–19.1, 202)	**<0.001**
Eaves closed	27.8 (21.6–33.9, 980)	10.0 (6.5–13.5, 1364)	**<0.001**
Mosquito prevention methods			
House sprayed (%)	1.3 (0.0–3.3, 973)	0.0 (0.0–0.0, 1364)	—-
Mosquito coils, insecticide spray,			
repellents used in prior week	15.6 (10.6–20.7, 1016)	11.9 (8.4–15.5, 1423)	0.285
At least one bednet in house (%)	79.3 (74.6–84.0, 1016)	71.5 (66.4–76.7, 1423)	0.159
Slept under any net the prior night			
<5	71.4 (62.8–80.1, 175)	60.0 (52.6–67.4, 250)	0.075
5–14	55.4 (46.7–64.2, 285)	39.6 (32.7–46.5, 422)	**0.007**
≥15	67.7 (62.3–73.0, 501)	58.9 (54.4–63.3, 647)	0.055
Overall	64.7 (59.5–70.0, 961)	52.9 (48.3–57.5, 1319)	**0.008**
Slept under an ITN the prior night			
<5	55.4 (46.4–64.5, 175)	40.8 (33.5–48.1, 250)	**0.012**
5–14	36.6 (28.7–44.6, 284)	24.2 (18.4–29.9, 422)	**0.007**
≥15	46.7 (41.2–52.2, 501)	37.7 (33.3–42.2, 647)	**0.020**
Overall	45.3 (40.1–50.5, 960)	34.0 (29.8–38.1, 1319)	**0.001**
Fever in prior 24 hours			
<5	32.9 (25.6–40.3, 173)	28.6 (22.2–34.9, 259)	0.307
5–14	21.0 (15.6–26.5, 295)	16.5 (12.4–20.6, 442)	0.179
≥15	38.6 (34.1–43.2, 515)	31.5 (27.5–35.4, 658)	**0.043**
Overall	32.3 (28.5–36.2, 983)	26.0 (22.7–29.4, 1359)	**0.035**
Took antimalarial prior 2 weeks			
<5	14.4 (9.2–19.7, 180)	11.7 (7.4–16.0, 265)	0.372
5–14	10.1 (6.4–13.8, 306)	10.3 (6.9–13.7, 466)	0.914
≥15	9.1 (6.4–11.7, 530)	13.9 (11.1–16.7, 692)	**0.031**
Overall	10.3 (8.1–12.5, 1016)	12.3 (10.1–14.5, 1423)	0.305
Took ACT prior 2 weeks			
<5	5.0 (1.5–8.5, 180)	1.5 (0.4–3.0, 265)	0.075
5–14	2.0 (0.2–3.7, 306)	1.7 (0.5–2.9, 466)	0.813
≥15	2.6 (1.2–4.1, 530)	2.0 (0.9–3.1, 692)	0.491
Overall	2.9 (1.6–4.1, 1016)	1.8 (1.1–2.6, 1423)	0.097

The second survey (post IRS-1) was conducted at the end of the low malaria transmission season and just before the short rainy season. Univariate analyses showed fewer differences between the two populations, with several related to the IRS (Table 2). The median elevation of compounds interviewed in the IRS district was higher than that of the non-IRS district (1406 m vs. 1260 m, p<0.001). However, there was no difference in the proportion of persons surveyed living above 1500 m. Differences in the types of houses in which participants resided were noted, but among respondents in this survey, residents of the IRS district were less likely than those in the non-IRS district to live in semi-permanent houses (60.6% vs 67.2%, p = 0.030) and more likely to live in permanent houses (22.4% vs 14.0%, p = 0.030). Residents of the IRS district were more likely to have reported their house was sprayed in the last 12 months (p<0.001), although coverage was only 65.9%, lower than the programmatic goal of 85%. There was no difference in reported rates of net usage in the two districts. There were no significant differences in the use of anti-malarials. Overall, persons living in the IRS district were less likely to have reported use of an ACT (1.5% vs. 4.5%, p<0.001) although when broken down by age, only among children <5 years of age were residents of the IRS district significantly less likely to have reported use of an ACT in the previous 2 weeks (0.6% vs. 7.7%, p = 0.001).

**Table 2:** Characteristics of the survey population during the 1^st^ follow up survey (November 2008). Values are presented with 95% CI and sample size in parentheses. P-values represent univariate comparisons between IRS and non-IRS districts. Comparisons that were statistically significant at p<0.05 are indicated in bold. All comparisons controlled for clustering within enumeration areas.

	Non-IRS District	IRS District	P value
N	1366	1588	
Median age in years (IQR)	14.3 (6.4–31.4)	13.9 (6.2–31.4)	0.452
Female	57.2 (54.9–59.6, 1366)	55.9 (53.5–58.2, 1588)	0.458
Caretaker with some secondary education (for children <5 years)	28.7 (20.2–37.1, 272)	24.1 (17.4–30.8, 328)	0.769
Living with biological mother (children <5 only)	95.3 (92.6–97.9, 274)	94.5 (91.6–97.5, 328)	0.662
Median elevation (m) (IQR)	1260 (1206–1417)	1406 (1285–1501)	**<0.001**
Elevation ≥1500 m	13.6 (9.0–18.2, 1366)	27.6 (21.5–33.6, 1588)	0.322
Household type			
Traditional mud hut	18.9 (14.2–23.5, 255)	17.0 (12.6–21.4, 268)	
Semi-permanent (corrugated iron roof)	67.2 (61.8–72.5, 908)	60.6 (55.4–65.9, 957)	
Permanent (concrete or stone walls)	14.0 (10.2–17.8, 189)	22.4 (17.6–27.2, 353)	**0.030**
Eaves closed	12.8 (8.8–16.8, 1364)	10.5 (7.1–13.9, 1581)	0.679
Mosquito prevention methods			
House sprayed (%)	0.4 (0.0–1.1, 1364)	65.9 (60.5–71.2, 1573)	**<0.001**
Mosquito coils, insecticide spray, repellents used in prior week	7.5 (4.4–10.6, 1366)	8.4 (4.5–12.2, 1588)	0.723
At least one bednet in house (%)	56.4 (51.6–61.3, 1366)	58.2 (53.8–62.7, 1588)	0.947
Slept under any net the prior night			
<5	63.7 (55.6–71.9, 273)	67.7 (60.6–74.8, 328)	0.561
5–14	44.1 (37.1–51.2, 435)	44.1 (37.6–50.7, 494)	0.558
≥15	62.8 (57.9–67.6, 642)	64.2 (59.8–68.7, 758)	0.932
Overall	57.0 (52.1–61.8, 1350)	58.7 (54.2–63.1, 1580)	0.996
Slept under an ITN the prior night			
<5	43.2 (35.8–50.7, 273)	50.0 (42.6–57.4, 328)	0.349
5–14	27.6 (21.8–33.4, 435)	31.0 (24.9–37.1, 494)	0.653
≥15	39.3 (34.7–43.8, 642)	44.9 (40.1–49.6, 758)	0.304
Overall	36.3 (31.9–40.7, 1350)	41.6 (37.0–46.1, 1580)	0.261
Fever in prior 24 hours			
<5	24.9 (18.7–31.1, 273)	19.8 (14.3–25.3, 328)	0.113
5–14	14.1 (10.3–17.8, 434)	10.3 (6.6–14.0, 486)	0.119
≥15	24.3 (20.5–28.1, 641)	21.8 (18.7–24.9, 758)	0.303
Overall	21.1 (18.2–24.1, 1348)	17.8 (15.2–20.4, 1572)	0.095
Took antimalarial prior 2 weeks			
<5	15.3 (10.8–19.9, 274)	10.1 (6.6–13.5, 328)	0.053
5–14	8.0 (5.1–10.9, 438)	7.8 (4.7–11.0, 498)	0.787
≥15	10.4 (8.0–12.8, 654)	10.6 (8.2–13.0, 762)	0.988
Overall	10.6 (8.7–12.5, 1366)	9.6 (7.5–11.7, 1588)	0.350
Took ACT prior 2 weeks			
<5	7.7 (4.3–11.1, 274)	0.6 (0.0–1.5, 328)	**0.001**
5–14	3.9 (1.7–6.1, 438)	1.8 (0.4–3.2, 498)	0.104
≥15	3.7 (2.2–5.2, 654)	1.7 (0.7–2.7, 762)	0.072
Overall	4.5 (3.1–5.9, 1366)	1.5 (0.8–2.2, 1588)	**<0.001**

During the third survey (post IRS-2), no differences were found in house type or the proportion of houses with open eaves (Table 3). Consistent with the prior surveys, participants living in the IRS district were found to live on average at higher elevations than those of the non-IRS district (1378 m vs. 1173 m, p<0.001) although there were no differences in the proportion of people living above 1500 m. The residents of the non-IRS district were more likely to have used mosquito coils or repellents (13.1% vs 6.3%, p = 0.022) while residents of the IRS district were more likely to report that their house had been sprayed with IRS in the last 12 months (60.5% vs 1.4%, p<0.001). Consistent with the post IRS-1 results, household IRS coverage was less than the coverage goal of 85% set by the National Malaria Control Program. Use of any net and use of an ITN was similar in both districts among all age groups. There were no differences in reported use of anti-malarials among any age group. However, reported ACT use was generally higher in the IRS district although the difference was statistically significant only among children aged 5 to 14 years of age (1.9% in the non-IRS district vs. 4.1% in the IRS district, p = 0.046).

**Table 3:** Characteristics of the survey population during the 2^nd^ post-IRS survey (August 2009). Values are presented with 95% CI and sample size in parentheses. P-values represent univariate comparisons between IRS and non-IRS districts. Comparisons that were statistically significant at p<0.05 are indicated in bold. All comparisons controlled for clustering within enumeration areas.

	Non-IRS District	IRS District	P value
N	1738	1742	
Median age in years (IQR)	14.2 (6.5–29.6)	14.0 (6.7–28.1)	0.508
Female	56.2 (54.0–58.5, 1738)	55.6 (53.2–58.0, 1742)	0.702
Caretaker with some secondary education (for children <5 years)	25.2 (17.0–33.4, 322)	31.0 (23.3–38.7, 316)	0.241
Living with biological mother (children <5 only)	94.4 (91.5–97.4, 322)	91.8 (88.1–95.4, 316)	0.676
Median elevation (m) (IQR)	1173 (1149–1302)	1378 (1192–1465)	**<0.001**
Elevation >1500 m	9.7 (6.1–13.4, 1738)	15.4 (10.6–20.3, 1742)	0.681
House type^1^			
Traditional mud hut	13.0 (9.6–16.5, 226)	11.1 (7.6–14.5, 193)	
Semi-permanent (corrugated iron roof)	71.0 (66.1–75.9, 1231)	70.8 (66.0–75.7, 1233)	
Permanent (concrete or stone walls)	16.0 (11.9–20.0, 277)	18.1 (14.0–22.2, 315)	0.616
Eaves closed	8.3 (5.4–11.2, 1738)	13.0 (9.4–16.6, 1742)	0.172
Mosquito prevention methods			
House sprayed (%)	1.4 (0.1–2.7, 1735)	60.5 (54.6–66.3, 1725)	**<0.001**
Mosquito coils, insecticide spray, repellents used in prior week	13.1 (9.5–16.6, 1738)	6.3 (3.8–8.7, 1742)	**0.022**
At least one bednet in house (%)	59.2 (54.8–63.7, 1738)	57.7 (53.3–62.2, 1742)	0.365
Slept under any net the prior night			
<5	68.1 (61.7–74.5, 320)	58.9 (52.1–65.7, 314)	0.067
5–14	48.9 (42.7–55.0, 575)	42.6 (36.4–48.8, 610)	0.116
≥15	61.0 (56.6–65.4, 834)	62.8 (58.5–67.2, 802)	0.859
Overall	58.3 (54.0–62.5, 1729)	55.0 (50.7–59.3, 1726)	0.158
Slept under an ITN the prior night			
<5	43.4 (36.7–50.2, 320)	39.8 (33.1–46.5, 314)	0.442
5–14	31.8 (25.8–37.9, 575)	31.0 (25.3–36.7, 610)	0.820
≥15	37.1 (32.6–41.5, 834)	43.9 (39.2–48.6, 802)	0.106
Overall	36.5 (32.2–40.8, 1729)	38.6 (34.3–42.9, 1726)	0.750
Fever in prior 24 hours			
<5	33.5 (27.6–39.5, 319)	30.9 (24.8–36.9, 314)	0.506
5–14	16.4 (12.8–20.1, 572)	15.7 (12.1–19.2, 606)	0.784
≥15	24.1 (20.9–27.4, 833)	29.5 (25.9–33.2, 799)	0.092
Overall	23.3 (20.7–25.9, 1724)	24.9 (22.1–27.7, 1719)	0.620
Took antimalarial prior 2 weeks			
<5	14.9 (10.6–19.2, 322)	9.8 (6.2–13.4, 316)	**0.042**
5–14	10.6 (7.5–13.8, 575)	8.9 (6.5–11.4, 615)	0.310
≥15	12.1 (9.7–14.5, 841)	10.2 (8.0–12.5, 811)	0.303
Overall	12.1 (10.1–14.2, 1738)	9.7 (8.0–11.4, 1742)	0.061
Took ACT prior 2 weeks			
<5	4.3 (2.0–6.7, 322)	4.7 (2.1–7.4, 316)	0.796
5–14	1.9 (0.7–3.1, 575)	4.1 (2.5–5.6, 615)	**0.046**
≥15	3.0 (1.9–4.1, 841)	2.8 (1.7–4.0, 811)	0.833
Overall	2.9 (2.0–3.7, 1738)	3.6 (2.6–4.6, 1742)	0.290

### Impact of IRS on malaria-related outcomes in an area with moderate ITN coverage

**Fig 3:**
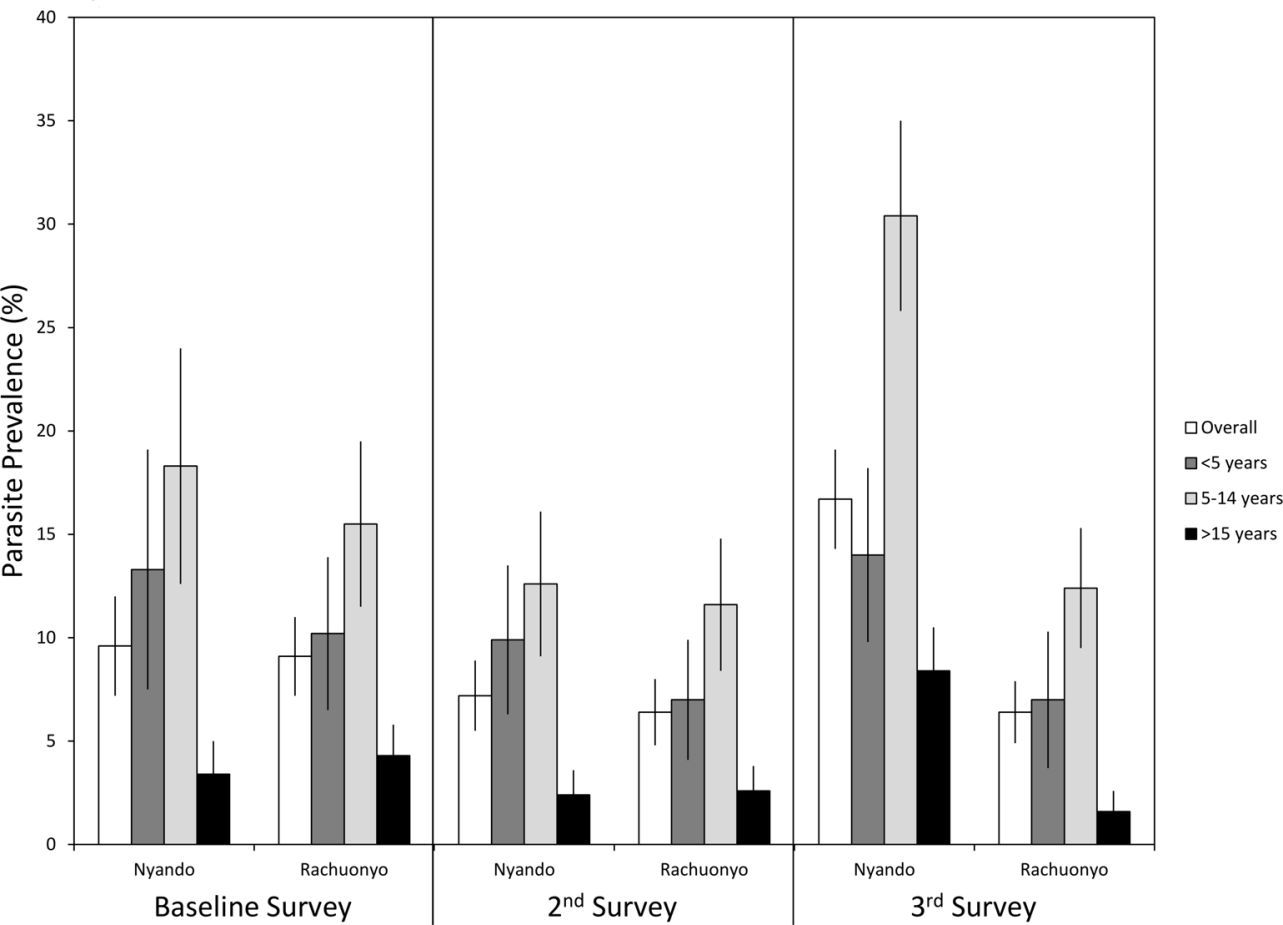
Prevalence of malaria parasitemia by age survey and district. Error bars represent 95% confidence limits.

**Fig 4:**
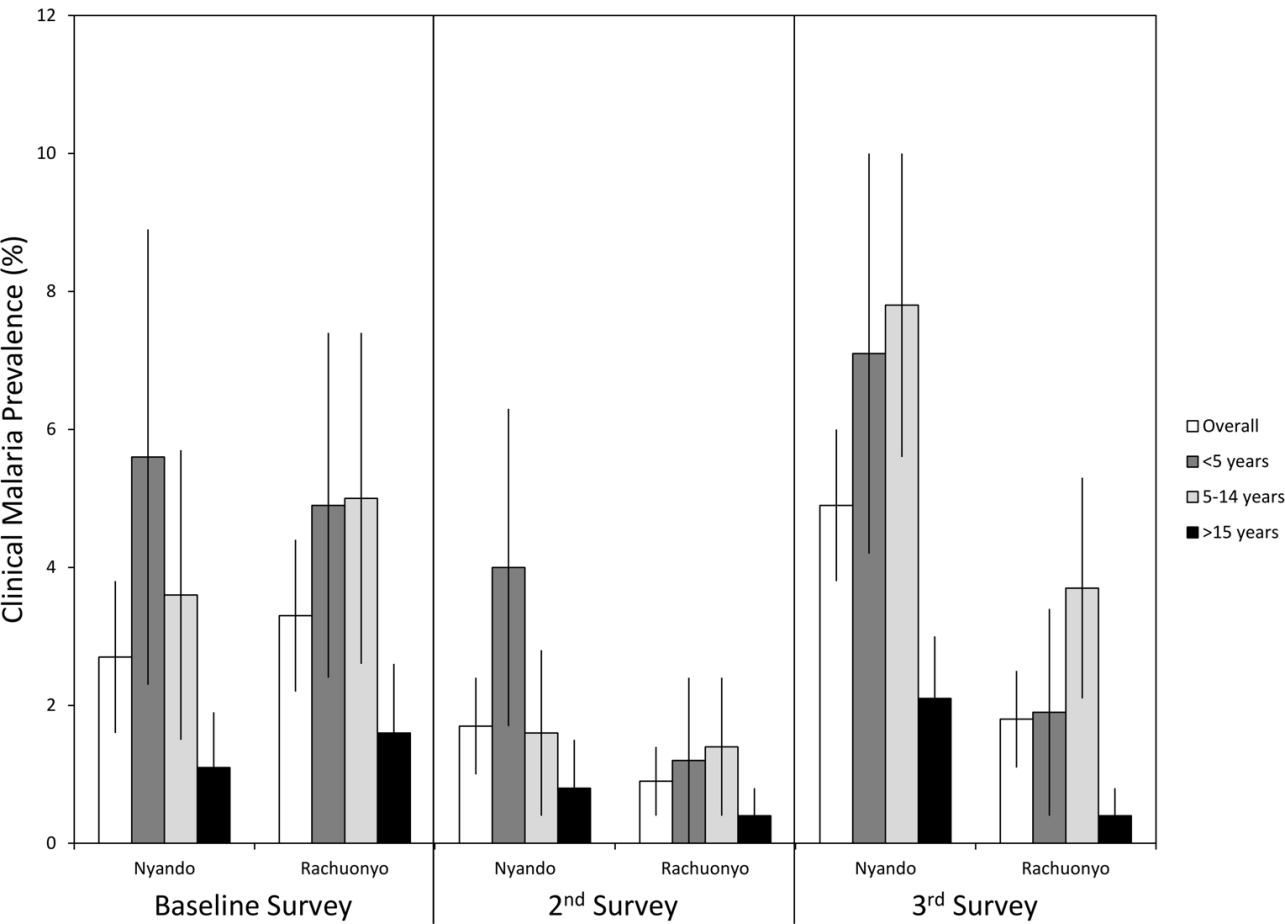
Prevalence of clinical malaria by age, survey and district. Error bars represent 95% confidence limits.

**Fig 5:**
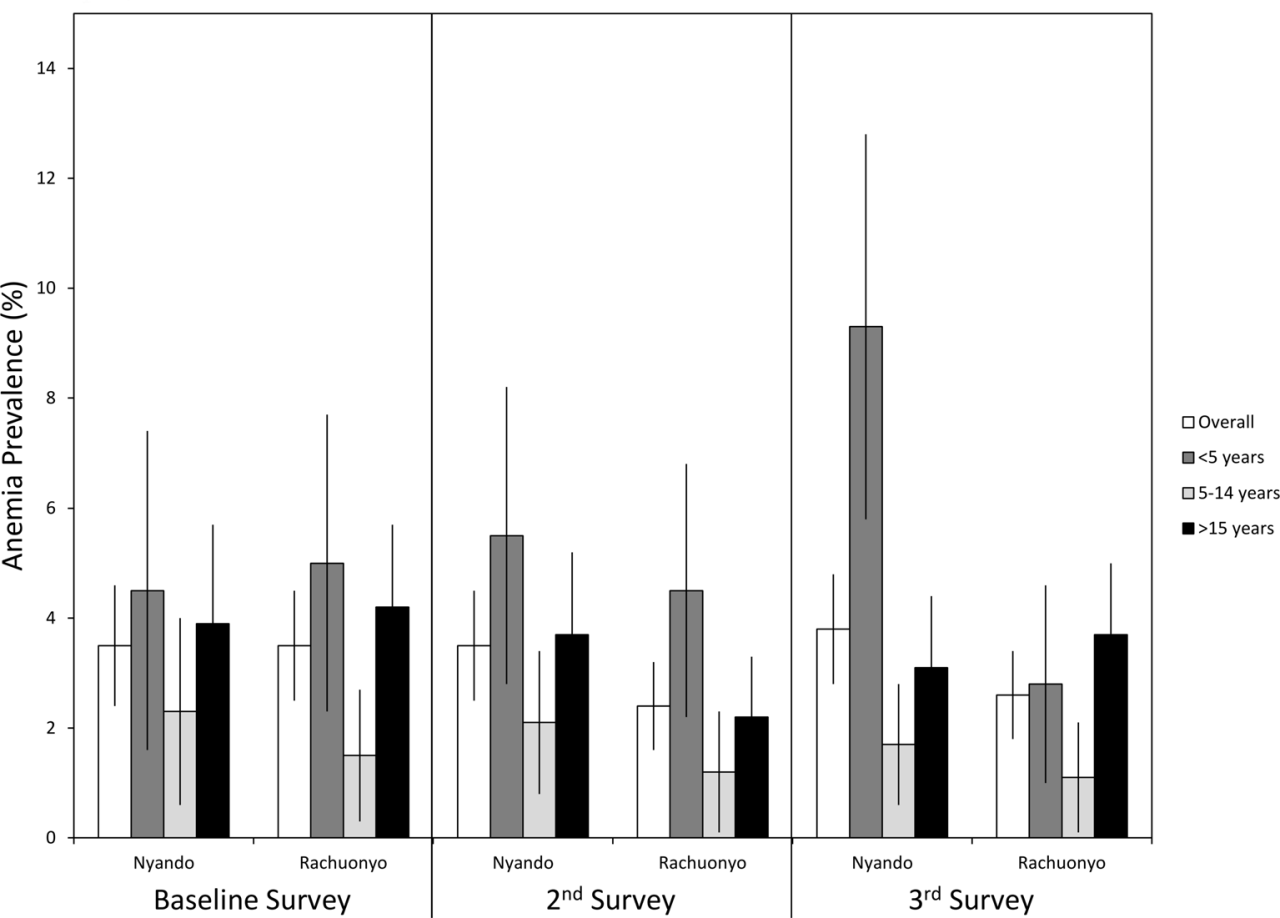
Prevalence of anemia by age, survey and district. Rachuonyo district received one round of IRS before the 2^nd^ survey and another round of IRS before the 3^rd^ survey. Error bars represent 95% confidence limits.

The prevalence of malaria parasitemia, clinical malaria, and anemia along with mean parasite densities and hemoglobin levels during each survey are provided in Table 4 and shown in Figs 3, 4 and 5. Overall parasite prevalence by microscopy was 9.6% in the non-IRS district and 9.1% in the IRS district before IRS was conducted (p = 0.817), while the geometric mean parasite densities in children aged 5 to 14 years were higher in the IRS district (1,349 versus 340 parasites per microliter, p = 0.004). Overall parasite densities were also higher in the IRS district at baseline (1,075 versus 481 parasites per microliter, p = 0.022). There were no differences in the prevalences of clinical malaria, mean hemoglobin levels or anemia defined as Hb<8 g/dL between the two districts at baseline.

**Table 4:** Prevalence of malaria related outcomes by survey and district. Values in bold are significantly different between IRS and non-IRS districts at a<0.05 in univariate analyses. All comparisons controlled for clustering within compounds and within enumeration areas.

	Baseline		Post IRS-1		Post IRS-2	
	Non-IRS District	IRS District	Non-IRS District	IRS District	Non-IRS District	IRS District
*P*. *falciparum* parasitemia prevalence						
<5	13.3 (7.5–19.2, 180)	10.2 (6.5–13.9, 265)	9.9 (6.3–13.4, 274)	7.0 (4.1–9.9, 328)	14.0 (9.8–18.2, 322)	7.0 (3.7–10.2, 316)
5–14	18.3 (12.6–24.0, 306)	15.5 (11.5–19.4, 466)	12.6 (9.1–16.1, 438)	11.6 (8.4–14.9, 498)	**30.4 (25.8–35.1, 575)**	**12.4 (9.5–15.2, 615)**
≥15	3.4 (1.8–5.0, 530)	4.3 (2.8–5.9, 692)	2.4 (1.2–3.7, 654)	2.6 (1.4–3.8, 762)	**8.4 (6.3–10.6, 841)**	**1.6 (0.6–2.6, 811)**
Overall	9.6 (7.2–12.0, 1016)	9.1 (7.2–10.9, 1423)	7.2 (5.5–8.8, 1366)	6.4 (4.8–7.9, 1588)	**16.7 (14.3–19.2, 1738)**	**6.4 (4.9–7.8, 1742)**
Geometric mean *P*. *falciparum* parasite density (per μl) among those with parasites						
<5	1654 (533–5135, 24)	2606 (971–6,994, 26)	2497 (936–6662, 27)	603 (201–1808, 23)	3331 (1812–6122, 44)	1763 (624–4984, 22)
5–14	**340 (178–649, 56)**	**1349 (832–2186, 72)**	424 (241–748, 55)	335 (230–487, 57)	501 (382–657, 173)	478 (312–732, 76)
≥15	273 (79–942, 18)	289 (133–630, 30)	295 (114–761, 16)	130 (57–292, 20)	267 (188–380, 70)	210 (64–691, 13)
Overall	**481 (287–805, 98)**	**1,075 (723–1597, 128)**	**652 (410–1034, 98)**	**317 (220–457, 100)**	575 (459–719, 287)	562 (382–828, 111)
Clinical malaria (*P*. *falciparum* parasitemia with fever in the last 24 hours)						
<5	5.6 (2.3–9.0, 178)	4.9 (2.4–7.5, 264)	**4.0 (1.7–6.3, 274)**	**1.2 (0.0–2.4, 328)**	**7.1 (4.2–10.1, 322)**	**1.9 (0.4–3.4, 316)**
5–14	3.6 (1.5–5.7, 303)	5.0 (2.6–7.4, 460)	1.6 (0.4–2.8, 438)	1.4 (0.4–2.4, 498)	**7.8 (5.6–10.1, 575)**	**3.7 (2.1–5.4, 615)**
≥15	1.1 (0.3–2.0, 530)	1.6 (0.6–2.6, 692)	0.8 (0.1–1.4, 654)	0.4 (0.0–0.8, 762)	**2.1 (1.2–3.1, 841)**	**0.4 (0.0–0.8, 810)**
Overall	2.7 (1.6–3.8, 1011)	3.3 (2.2–4.4, 1416)	1.7 (1.0–2.4, 1366)	0.9 (0.4–1.3, 1588)	**4.9 (3.8–6.1, 1738)**	**1.8 (1.1–2.6, 1741)**
Mean hemoglobin (g/dL)						
<5	10.7 (10.5–11.0, 176)	10.8 (10.6–11.0, 259)	**10.7 (10.6–10.9, 274)**	**11.4 (11.2–11.5, 328)**	**10.4 (10.3–10.6, 322)**	**11.3 (11.1–11.4, 316)**
5–14	12.2 (12.0–12.4, 298)	12.3 (12.2–12.5, 462)	**12.3 (12.2–12.5, 437)**	**12.9 (12.8–13.1, 498)**	**12.2 (12.1–12.4, 573)**	**12.8 (12.7–12.9, 615)**
≥15	12.8 (12.6–13.0, 519)	12.7 (12.5–12.9, 685)	**12.9 (12.7–13.1, 654)**	**13.3 (13.1–13.4, 762)**	**12.8 (12.6–12.9, 841)**	**13.1 (12.9–13.3, 811)**
Overall	12.2 (12.1–12.4, 993)	12.2 (12.1–12.3, 1406)	**12.3 (12.2–12.4, 1365)**	**12.8 (12.7–12.9, 1588)**	**12.2 (12.1–12.3, 1736)**	**12.7 (12.6–12.8, 1742)**
Anemia (HB<8 g/dL)						
<5	4.5 (1.6–7.5, 176)	5.0 (2.3–7.7, 259)	5.5 (2.8–8.1, 274)	4.5 (2.2–6.9, 328)	**9.3 (5.8–12.9, 322)**	**2.8 (1.0–4.7, 316)**
5–14	2.3 (0.6–4.1, 298)	1.5 (0.3–2.8, 462)	2.1 (0.8–3.4, 437)	1.2 (0.1–2.3, 498)	1.7 (0.6–2.9, 573)	1.1 (0.1–2.2, 615)
≥15	3.9 (2.1–5.6, 519)	4.2 (2.7–5.7, 685)	3.7 (2.2–5.1, 654)	2.2 (1.1–3.3, 762)	3.1 (1.8–4.3, 841)	3.7 (2.4–5.0, 811)
Overall	3.5 (2.4–4.7, 993)	3.5 (2.5–4.5, 1406)	3.5 (2.5–4.5, 1365)	2.4 (1.6–3.2, 1588)	3.8 (2.8–4.8, 1736)	2.6 (1.8–3.4, 1742)

During the post IRS-1 survey, no difference was found in parasite prevalence (6.4% in the IRS district vs 7.2% the non-IRS district, p = 0.718) or the prevalence of clinical malaria (0.9% in the IRS district vs. 1.7% in the non-IRS district, p = 0.076). Geometric mean parasite densities were lower overall (317 vs. 652 parasites per microliter, p = 0.022) in the IRS district. Mean hemoglobin levels were significantly higher in all age groups in the IRS district (overall Hb levels were 12.8 g/dL in the IRS district vs 12.3 g/dL in the non-IRS district, p<0.001) and the overall prevalence of anemia was lower in the IRS district (2.4% in the IRS district vs 3.6% in the non-IRS district) but there were no differences in the prevalence of anemia overall or within specific age categories.

Despite lower than desired IRS coverage, several indicators showed that IRS had a significant impact on malaria related indices by the time of the post IRS-2 survey (Table 4). Overall prevalence was 16.7% in the non-IRS district compared with 6.4% in the IRS district (p<0.001). The prevalence of clinical malaria (4.9% vs 1.8%, p = 0.001) and anemia in children <5 years of age (9.3% vs. 2.8%, p<0.006) were significantly lower in the IRS district. Mean hemoglobin was higher in the IRS district for all age categories. However, there were no differences in the geometric mean parasite densities between the IRS district and the non-IRS district.

### Multivariate models of malaria parasitemia

**Fig 6:**
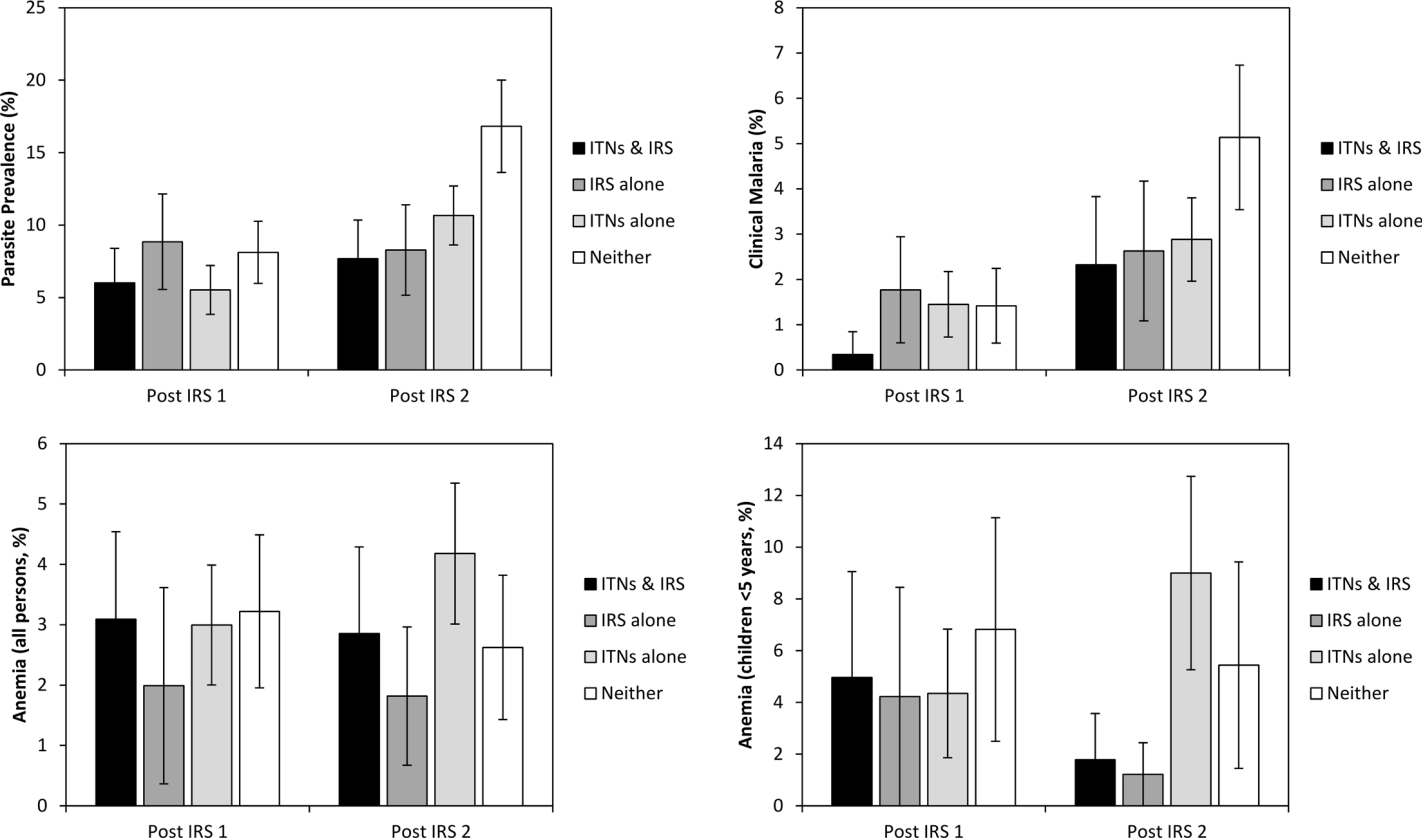
Prevalence of malaria parasitemia, clinical malaria, anemia and the prevalence of anemia in children <5 years of age by survey. Survey respondents were categorized as those who received IRS in the previous 12 months and used an ITN the previous night, those who received IRS alone, those who used an ITN the previous night but did not receive IRS and among those who neither used an ITN the previous night nor received IRS in the 12 months prior to the survey. Error bars represent 95% confidence limits.

When vector control was considered as a categorical variable with 4 levels, the impact of ITNs and IRS on the prevalence of malaria parasitemia measured at the time of the post IRS-1 survey, conducted 3 months after the first IRS round, was minimal (Fig 6, Table 5). No significant differences were found in parasite prevalence between users of ITNs only (prevalence = 5.5%) or recipients of IRS only (prevalence = 8.8%), those with both ITNs and IRS (prevalence = 6.0%) and those who neither used ITNs nor received IRS (prevalence = 8.1%). Elevation (OR = 0.43, 95% CI = 0.20–0.94, p = 0.034) and living in a permanent house (OR = 0.27, 95% CI = 0.09–0.77, p = 0.013) were associated with lower risks of malaria while children <5 years (OR = 3.59, 95% CI = 2.33–5.54, p<0.001) and children aged 5 to 14 years (OR = 5.07, 95% CI = 3.38–7.59, p<0.001) had lower odds of malaria parasitemia compared with adults.

**Table 5:** Odds ratios from a multivariate regression model of the impact of different factors on infection with *P*. *falciparum* malaria parasitemia, clinical malaria and anemia. Vector control (ITNs, IRS, both or neither) was included as a single categorical variable in all models. Comparisons that were statistically significant a p<0.05 are indicated in bold.

	*P. falciparum* parasitemia		Clinical malaria		Anemia (Hb<8 g/dL)	
Parameter	Post IRS 1	Post IRS 2	Post IRS 1	Post IRS 2	Post IRS 1	Post IRS 2
IRS & ITNs	0.89 (0.47–1.69)	**0.52 (0.28–96)**	0.14 (0.02–1.09)	0.58 (0.25–1.36)	0.76 (0.37–1.56)	0.84 (0.43–1.62)
IRS alone	1.37 (0.73–2.58)	**0.44 (0.24–0.80)**	1.71 (0.61–4.78)	**0.47 (0.24–0.93)**	0.56 (0.20–1.54)	0.71 (0.32–1.56)
ITNs alone	0.71 (0.41–1.23)	**0.68 (0.49–0.93)**	0.95 (0.44–2.05)	**0.59 (0.36–0.97)**	0.66 (0.39–1.12)	1.10 (0.60–2.01)
Neither	Ref.	Ref.	Ref.	Ref.	Ref.	Ref.
Elevation ≥1500	**0.43 (0.20–0.94)**	**0.25 (0.10–0.61)**	0.65 (0.23–1.82)	NA	0.54 (0.18–1.63)	0.42 (0.16–1.13)
Elevation <1500	Ref.	Ref.	Ref.	Ref.	Ref.	Ref.
Gender (Female)	1.07 (0.80–1.42)	1.09 (0.87–1.35)	0.95 (0.49–1.82)	**1.99 (1.34–2.96)**	1.27 (0.78–2.05)	1.18 (0.80–1.75)
Gender (Male)	Ref.	Ref.	Ref.	Ref.	Ref.	Ref.
Age, <5 years	**3.59 (2.33–5.54)**	**2.40 (1.72–3.35)**	**4.84 (1.87–12.5)**	**4.24 (2.61–6.88)**	1.76 (0.90–3.43)	**1.81 (1.10–2.97)**
Age, 5 to 14 years	**5.07 (3.38–7.59)**	**5.42 (4.25–6.91)**	**2.34 (1.14–4.82)**	**6.14 (3.64–10.4)**	0.55 (0.29–1.04)	**0.39 (0.18–0.84)**
Age, ≥15 years	Ref.	Ref.	Ref.	Ref.	Ref.	Ref.
History of ACT use (Yes)	2.07 (0.88–4.84)	**0.35 (0.15–0.81)**	**4.11 (1.69–10.0)**	1.13 (0.43–2.94)	1.89 (0.60–5.99)	2.09 (0.95–4.59)
History of ACT use (No)	Ref.	Ref.	Ref.	Ref.	Ref.	Ref.
Use coils or sprays (Yes)	0.48 (0.18–1.28)	**1.71 (1.17–2.49)**	0.88 (0.20–3.81)	1.09 (0.53–2.23)	0.59 (0.18–1.88)	1.67 (0.87–3.23)
Use coils or sprays (No)	Ref.	Ref.	Ref.	Ref.	Ref.	Ref.
House type, Permanent	**0.27 (0.09–0.77)**	**0.31 (0.16–0.58)**	0.25 (0.05–1.18)	**0.25 (0.11–0.57)**	0.81 (0.35–1.87)	1.05 (0.53–2.10)
House type, Semi-permanent	0.78 (0.44–1.36)	0.67 (0.44–1.03)	0.52 (0.24–1.11)	**0.50 (0.30–0.82)**	0.67 (0.35–1.30)	0.81 (0.48–1.36)
House type, Traditional	Ref.	Ref.	Ref.	Ref.	Ref.	Ref.

By the post IRS-2 survey, prevalence of parasitemia was lowest among those who had both ITNs and IRS (7.7%), was slightly higher among those who received IRS but did not use ITNs (8.3%), and was slightly higher again among those who used ITNs but did not receive IRS (10.7%). Prevalence was 16.8% among persons with neither IRS nor ITNs. At the post IRS-2 survey, the prevalence of malaria infection was significantly lower among those who had either ITNs (OR = 0.68, 95% CI = 0.49–0.93, p = 0.016) or IRS (OR = 0.44, 95% CI = 0.24–0.80, p = 0.008). Those who had both were less like to have malaria parasites (OR = 0.52, 95% CI = 0.28–0.96, p = 0.036) compared to those who had neither ITNs nor IRS. Elevation (OR = 0.25, 95% CI = 0.10–0.61, p = 0.002), a history of ACT use during the prior two weeks (OR = 0.35, 95% CI = 0.15–0.81, p = 0.014) and residence in a permanent house (OR = 0.31, 95% CI = 0.16–0.58, p<0.001) were associated with reduced risk of malaria infection. Children <5 years of age (OR = 2.40, 95% CI = 1.72–3.35, p<0.001) and children 5 to 14 years of age (OR = 5.42, 95% CI = 4.25–6.91, p<0.001) were significantly more likely to be parasitemic compared with adults at the time of the post- IRS-2 survey.

**Fig 7:**
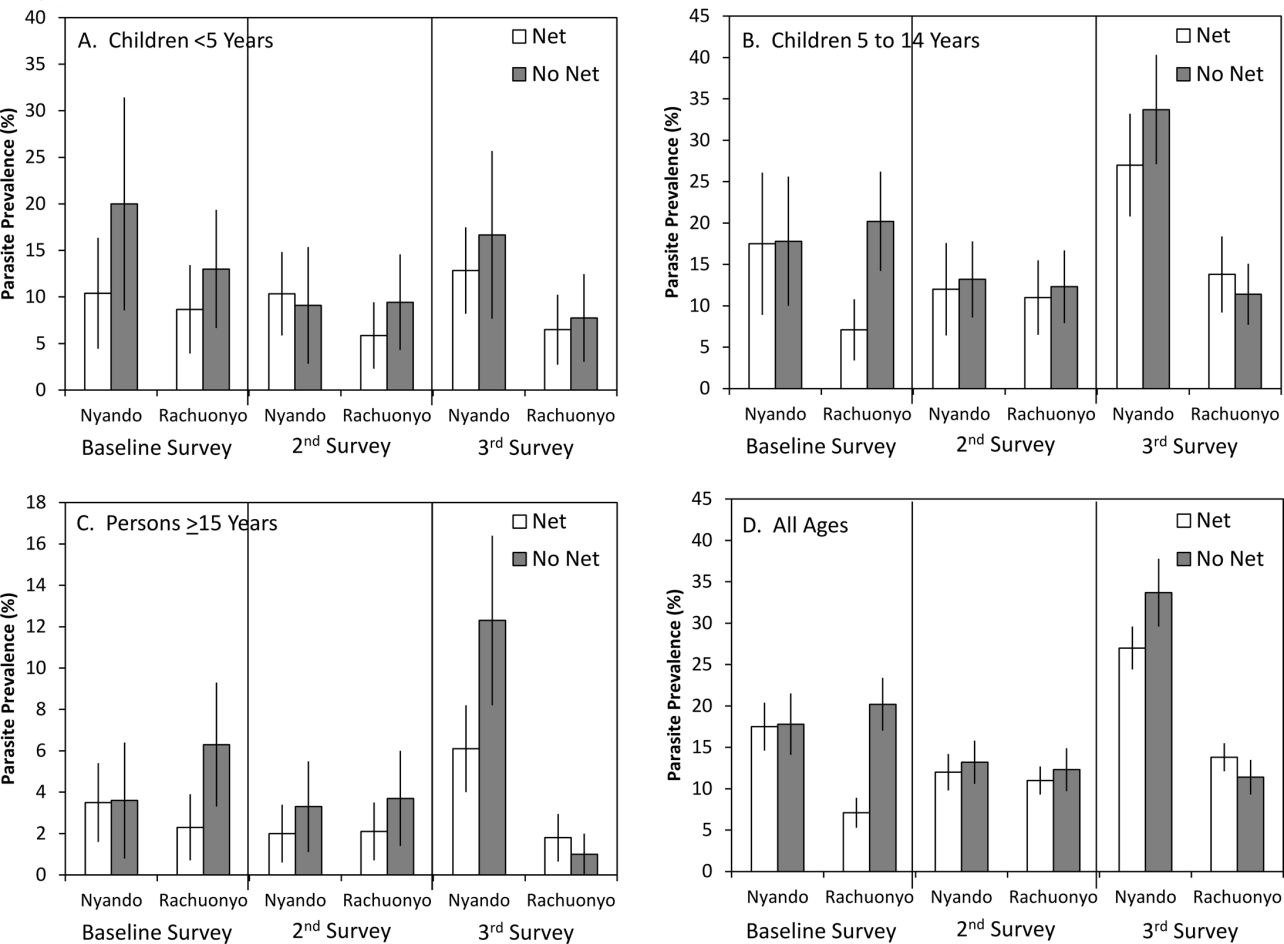
Prevalence of malaria parasitemia, clinical malaria, anemia and the prevalence of anemia in children <5 years of age by survey, district and ITN use the previous night.

Additional multivariate models incorporating an interaction between ITN use and district as a proxy for IRS were also assessed (Table 6). Malaria parasitemia prevalence by district and ITN use is shown in Fig 7. Separate models were run for each survey. At baseline, parasite prevalence was not associated with district or use of an ITN the previous night. There was a significant interaction between ITN use and district (OR = 0.37, 95% CI = 0.17–0.79, p = 0.011). In conditional estimates of the effects of ITNs, the use of an ITN was associated with a reduced odds of malaria parasitemia in the IRS district (OR = 0.29, 95% CI = 0.17–0.50, p<0.001) but not in the non-IRS district (OR = 0.80, 95% CI = 0.46–1.36, p = 0.406) (Table 7). Similarly, at baseline, the effect of district was conditional on ITN use with a statistically significant reduced risk of malaria parasitemia among net users in the district that would later receive IRS compared to net users in the non-IRS district (OR = 0.44, 95% CI = 0.23–0.85, p = 0.014). There was no difference among non-users between the two districts (OR = 1.19, 95% CI = 0.67–2.13, p = 0.549). The only other variable associated with malaria parasitemia at baseline was age with children <5 years (OR = 3.59, 95% CI = 2.13–6.05, p<0.001) and children aged 5 to 14 years (OR = 4.72, 95% CI = 3.07–7.25, p<0.001) having greater odds of malaria parasitemia compared to adults. During the post IRS-1 survey, neither district, the use of ITNs nor the interaction between these variables was statistically significant. Elevation was associated with lower odds of malaria parasitemia (OR = 0.44, 95% CI = 0.21–0.94, p = 0.033) as was living in a permanent house (OR = 0.27, 95% CI = 0.10–0.78, p = 0.015). Similar to the baseline survey, children <5 years (OR = 3.58, 95% CI = 2.33–5.52, p<0.001) and children aged 5 to 14 years (OR = 5.12, 95% CI = 3.40–7.69, p<0.001) had greater odds of malaria parasitemia compared to adults. Although the interaction term was not significant, use of a net in the IRS district was associated with a significant reduction in risk of malaria parasitemia (OR = 0.56, 95% CI = 0.36–0.88, p = 0.012) while use of a net in the non-IRS district was not (OR = 0.85, 95% CI = 0.45–1.62, p = 0.628).

**Table 6:** Multivariate regression model of the impact of different factors on infection with *P*. *falciparum* malaria parasitemia. The model included an interaction between net use and district (a proxy for IRS). Comparisons that were statistically significant a p<0.05 are indicated in bold.

	Baseline		Post IRS 1		Post IRS 2	
Parameter	OR	*p*	OR	*p*	OR	*p*
Rachuonyo (IRS)	1.19 (0.67, 2.13)	0.549	1.34 (0.71, 2.53)	0.374	**0.23 (0.12, 0.45)**	**<0.001**
Nyando (non-IRS)	1.00	Ref.	1.00	Ref.	1.00	Ref.
Use a net (Yes)	0.80 (0.46, 1.36)	0.406	0.85 (0.45, 1.62)	0.628	**0.63 (0.45, 0.88)**	**0.007**
Use a net (No)	1.00	Ref.	1.00	Ref.	1.00	Ref.
Rachuonyo * Use a net (Yes)	0.37 (0.17, 0.79)	0.011	0.66 (0.30, 1.43)	0.291	**1.86 (1.02, 3.39)**	**0.042**
Rachuonyo * Use a net (No)	1.00	Ref.	1.00	Ref.	1.00	Ref.
Nyando * Use a net (Yes)	1.00	Ref.	1.00	Ref.	1.00	Ref.
Nyando * Use a net (No)	1.00	Ref.	1.00	Ref.	1.00	Ref.
Elevation ≥1500	0.26 (0.05, 1.40)	0.117	**0.44 (0.21, 0.94)**	**0.033**	**0.24 (0.08, 0.69)**	**0.008**
Elevation <1500	1.00	Ref.	1.00	Ref.	1.00	Ref.
Gender (Female)	0.82 (0.55, 1.23)	0.339	1.07 (0.80, 1.43)	0.652	1.09 (0.88, 1.36)	0.419
Gender (Male)	1.00	Ref.	1.00	Ref.	1.00	Ref.
Age, <5 years	**3.59 (2.13, 6.05)**	**<0.001**	**3.58 (2.33, 5.52)**	**<0.001**	**2.39 (1.70, 3.37)**	**<0.001**
Age, 5 to 14 years	**4.72 (3.07, 7.25)**	**<0.001**	**5.12 (3.40, 7.69)**	**<0.001**	**5.64 (4.37, 7.28)**	**<0.001**
Age, ≥15 years	1.00	Ref.	1.00	Ref.	1.00	Ref.
History of ACT use (Yes)	1.02 (0.37, 2.77)	0.976	2.10 (0.89, 4.96)	0.090	**0.36 (0.15, 0.85)**	**0.020**
History of ACT use (No)	1.00	Ref.	1.00	Ref.	1.00	Ref.
Use coils or sprays (Yes)	0.68 (0.36, 1.28)	0.229	0.50 (0.19, 1.30)	0.155	**1.63 (1.09, 2.45)**	**0.019**
Use coils or sprays (No)	1.00	Ref.	1.00	Ref.	1.00	Ref.
House type, Permanent	0.53 (0.25, 1.13)	0.101	**0.27 (0.10, 0.78)**	**0.015**	**0.29 (0.16, 0.56)**	**<0.001**
House type, Semi-permanent	0.96 (0.62, 1.49)	0.865	0.79 (0.46, 1.38)	0.407	**0.63 (0.41, 0.97)**	**0.036**
House type, Traditional	1.00	Ref.	1.00	Ref.	1.00	Ref.

**Table 7:** Effect of using a net conditional on district and effect of district conditional on net use on the risk of infection with *P*. *falciparum* malaria parasitemia. Comparisons that were statistically significant a p<0.05 are indicated in bold.

	Baseline		Post IRS 1		Post IRS 2	
Parameter	OR	*p*	OR	*p*	OR	*p*
Net use conditional on district (non-IRS district):						
Use a net (Yes)	0.80 (0.46, 1.36)	0.406	0.85 (0.45, 1.62)	0.628	**0.63 (0.45, 0.88)**	**0.007**
Use a net (No)	1.00	Ref.	1.00	Ref.	1.00	Ref.
Net use conditional on district (IRS district):						
Use a net (Yes)	**0.29 (0.17, 0.50)**	**<0.001**	**0.56 (0.36, 0.88)**	**0.012**	1.17 (0.73, 1.87)	0.526
Use a net (No)	1.00	Ref.	1.00	Ref.	1.00	Ref.
District conditional on net use (Use a net = Yes)						
District (IRS)	**0.44 (0.23–0.85)**	**0.014**	0.88 (0.43–1.79)	0.723	**0.44 (0.25–0.76)**	**0.003**
District (non-IRS)	1.00	Ref.	1.00	Ref.	1.00	Ref.
District conditional on net use (Use a net = No)						
District (IRS)	1.19 (0.67–2.13)	0.549	1.34 (0.71–2.53)	0.374	**0.23 (0.12–0.45)**	**<0.001**
District (non-IRS)	1.00	Ref.	1.00	Ref.	1.00	Ref.

At the post IRS-72 survey, elevation (OR = 0.24, 95% CI = 0.08–0.69, p = 0.008), a history of ACT use (OR = 0.36, 95% CI = 0.15–0.85, p = 0.020), living in a permanent house (OR = 0.29, 95% CI = 0.16–0.56, p<0.001) and living in a semi-permanent house (OR = 0.63, 95% CI = 0.41–0.97, p = 0.036) were all associated with lower odds of malaria parasitemia. Children <5 years of age (OR = 2.39, 95% CI = 1.70–3.37, p<0.001) and children 5 to 14 years of age (OR = 5.64, 95% CI = 4.37–7.28, p<0.001) again had greater odds of malaria parasitemia compared to adults. In this survey, residents of the IRS district had lower odds of malaria parasitemia compared to the non-IRS district (OR = 0.23, 95% CI = 0.12–0.45, p<0.001). Users of ITNs had lower odds of malaria parasitemia compared to non-users (OR = 0.63, 95% CI = 0.45–0.88, p = 0.007). However, the interaction term between district and ITN use was significant (p = 0.042) and therefore, estimates of the effect of ITNs conditional on IRS and estimates of the effect of IRS conditional on ITN use were also included (Table 7). In estimates of the impact of ITNs on malaria parasitemia conditional on district, residents of the non-IRS district who used ITNs had lower odds of parasitemia compared to non-users (OR = 0.63, 95% CI = 0.45–0.88, p = 0.007). In the IRS district, there was no association between ITN use and malaria parasitemia (OR = 1.17, 95% CI = 0.73–1.87, p = 0.526). Conversely, when the effect of IRS was made conditional on net use, there was a statistically significant reduction in parasitemia regardless of whether ITNs were used (OR = 0.44, 95% CI = 0.25–0.76, p = 0.003) or not (OR = 0.23, 95% CI = 0.12–45, p<0.001) (Table 7).

In a separate model that included interactions between district and elevation, an impact of IRS on parasite prevalence was not observed in areas above 1500 m after 2 rounds of IRS. Parasite prevalence in highland areas of the non-IRS district was 2.4% compared with 5.2% in the highlands of the IRS district during the third survey; however, the difference was not statistically significant (OR = 2.43, 95% CI = 0.62–9.53, p = 0.201). In contrast, the prevalence of malaria in lowland areas was significantly lower in the IRS district (6.6%) compared to the non-IRS district (18.3%) (OR = 0.29, 95% CI = 0.17–0.50, p<0.001).

### Multivariate models of clinical malaria

When vector control was categorized as a 4 level variable, the prevalence of clinical malaria at post IRS-1 was 1.8% for those who had received only IRS, 1.5% for those who had used an ITN the previous night but did not receive IRS, 0.3% for those with both ITNs and IRS and 1.4% among those who had not received either intervention (Fig 3). In a logistic regression model with vector control as a 4 level, categorical variable (Table 5), there were no significant differences associated with the different vector control interventions at the post IRS-1 survey. The only variables associated with clinical malaria during the post IRS-1 survey were age with higher odds of clinical malaria among children <5 years of age (OR = 4.84, 95% CI = 1.87–12.5, p = 0.001), children 5 to 14 years of age (OR = 2.34, 95% CI = 1.14–4.82, p = 0.021) and a recent history of ACT use (OR = 4.11, 95% CI = 1.69–10.0, p = 0.002).

After 2 rounds of IRS in one district, the prevalence of clinical malaria was 2.6% among those who had received IRS only, 2.9% among those who had used an ITN the previous night but did not receive IRS, 2.3% among those with both interventions and 5.1% among those with neither. Respondents who had received IRS (OR = 0.47, 95% CI = 0.24–0.93, p = 0.030) or had used an ITN the previous night (OR = 0.59, 95% CI = 0.36–0.97, p = 0.038) had significantly lower odds of clinical malaria compared to persons who had neither received IRS nor used an ITN the previous night. Persons who slept in a house that had been sprayed and slept under an ITN the previous night had lower odds of clinical malaria but it was not significantly different from those who had received neither intervention (OR = 0.58, 95% CI = 0.25–1.36, p = 0.210). The prevalence of clinical malaria remained associated with age with higher odds among children <5 years of age (OR = 4.24, 95% CI = 2.61–6.88, p<0.001) and children aged 5 to 14 years (OR = 6.14, 95% CI = 3.64–10.4, p<0.001) compared to adults. In addition, females were had a higher odds of clinical malaria than males (OR = 1.99, 95% CI = 1.34–2.96, p = 0.001) while those who lived in a permanent (OR = 0.25, 95% CI = 0.11–0.57, p = 0.001) or semi-permanent (OR = 0.50, 95% CI = 0.30–0.82, p = 0.006) house had lower odds of clinical malaria compared to persons who lived in a traditional hut.

The prevalence of clinical malaria by district and ITN use is shown in Fig 7. In multivariate models that included interactions between ITNs and district as a proxy for IRS (Table 8), age and history of ACT use were associated with clinical malaria during the baseline survey. The odds of clinical malaria were higher for children <5 years (OR = 4.60, 95% CI = 1.94–10.9, p = 0.001) and for children aged 5 to 14 years (OR = 2.63, 95% CI = 1.21–5.72, p = 0.014) compared to adults. The odds of clinical malaria were also higher among those reporting a history of ACT use (OR = 4.15, 95% CI = 1.45–11.9, p = 0.008). At the post IRS-1 survey, age was again associated with clinical malaria with higher odds among children <5 years old (OR = 4.75, 95% CI = 1.85–12.2, p = 0.001) and for children aged 5 to 14 years (OR = 2.35, 95% CI = 1.15–4.81, p = 0.020) compared to adults. Neither district (OR = 2.27, 95% CI = 0.79–6.52, p = 0.128) nor ITN use the previous night (OR = 1.75, 95% CI = 0.77–3.96, p = 0.182) were associated with clinical malaria. However, the interaction between these variables was statistically significant (p = 0.002) and therefore, the effects of ITNs and IRS conditional on each other are reported. In measuring the association of ITNs with clinical malaria conditional on district of residence, respondents in the IRS district had lower odds of clinical malaria if they used an ITN the previous night (OR = 0.05, 95% CI = 0.01–0.41, p = 0.005). No association between ITN use and clinical malaria was observed for the residents of the non-IRS district (OR = 1.75, 95% CI = 0.77–3.96, p = 0.182). For IRS conditional on net use, the prevalence of clinical malaria was not significantly different between non-users of ITN in the two districts (OR = 2.27, 95% CI = 0.79–6.52, p = 0.128). For ITN users, the prevalence of clinical malaria was significantly lower in the IRS district (OR = 0.06, 95% CI = 0.01–0.48, p = 0.007) (Table 9).

**Table 8:** Multivariate regression model of the impact of different factors on clinical malaria. The model included an interaction between net use and district (a proxy for IRS). Comparisons that were statistically significant a p<0.05 are indicated in bold.

	Baseline		Post IRS 1		Post IRS 2	
Parameter	OR	*p*	OR	*p*	OR	*p*
Rachuonyo (IRS)	1.25 (0.51, 3.07)	0.626	2.27 (0.79, 6.52)	0.128	**0.29 (0.14, 0.61)**	**0.001**
Nyando (non-IRS)	1.00	Ref.	1.00	Ref.	1.00	Ref.
Use a net (Yes)	0.45 (0.19, 1.05)	0.065	1.75 (0.77, 3.96)	0.182	**0.50 (0.29, 0.86)**	**0.011**
Use a net (No)	1.00	Ref.	1.00	Ref.	1.00	Ref.
Rachuonyo * Use a net (Yes)	0.49 (0.13, 1.84)	0.293	**0.03 (0.00, 0.26)**	**0.002**	2.15 (0.73, 6.35)	0.166
Rachuonyo * Use a net (No)	1.00	Ref.	1.00	Ref.	1.00	Ref.
Nyando * Use a net (Yes)	1.00	Ref.	1.00	Ref.	1.00	Ref.
Nyando * Use a net (No)	1.00	Ref.	1.00	Ref.	1.00	Ref.
Elevation ≥1500	NA	NA	0.66 (0.23, 1.88)	0.433	NA	NA
Elevation <1500	NA	NA	1.00	Ref.	NA	NA
Gender (Female)	0.86 (0.47, 1.57)	0.628	0.94 (0.49, 1.81)	0.854	**2.01 (1.37, 2.94)**	**<0.001**
Gender (Male)	1.00	Ref.	1.00	Ref.	1.00	Ref.
Age, <5 years	**4.60 (1.94, 10.9)**	**0.001**	**4.75 (1.85, 12.2)**	**0.001**	**4.19 (2.59, 6.78)**	**<0.001**
Age, 5 to 14 years	**2.63 (1.21, 5.72)**	**0.014**	**2.35 (1.15, 4.81)**	**0.020**	**5.97 (3.55, 10.0)**	**<0.001**
Age, ≥15 years	1.00	Ref.	1.00	Ref.	1.00	Ref.
History of ACT use (Yes)	**4.15 (1.45, 11.9)**	**0.008**	**4.06 (1.71, 9.65)**	**0.002**	1.20 (0.45, 3.21)	0.713
History of ACT use (No)	1.00	Ref.	1.00	Ref.	1.00	Ref.
Use coils or sprays (Yes)	0.53 (0.17, 1.62)	0.265	0.94 (0.22, 4.00)	0.932	1.09 (0.53, 2.25)	0.807
Use coils or sprays (No)	1.00	Ref.	1.00	Ref.	1.00	Ref.
House type, Permanent	0.47 (0.15, 1.49)	0.197	0.24 (0.05, 1.16)	0.076	**0.33 (0.16, 0.69)**	**0.003**
House type, Semi-permanent	0.96 (0.51, 1.80)	0.907	0.50 (0.24, 1.06)	0.071	**0.50 (0.30, 0.85)**	**0.010**
House type, Traditional	1.00	Ref.	1.00	Ref.	1.00	Ref.

**Table 9:** Effect of using a net conditional on district and effect of district conditional on net use on the risk of clinical malaria. Comparisons that were statistically significant a p<0.05 are indicated in bold.

	Baseline		Post IRS 1		Post IRS 2	
Parameter	OR	*p*	OR	*p*	OR	*p*
Net use conditional on district (non-IRS district):						
Use a net (Yes)	0.45 (0.19, 1.05)	0.065	1.75 (0.77, 3.96)	0.182	**0.50 (0.29, 0.86)**	**0.011**
Use a net (No)	1.00	Ref.	1.00	Ref.	1.00	Ref.
Net use conditional on district (IRS district):						
Use a net (Yes)	**0.22 (0.08, 0.62)**	**0.005**	**0.05 (0.01, 0.41)**	**0.005**	1.08 (0.42, 2.78)	0.873
Use a net (No)	1.00	Ref.	1.00	Ref.	1.00	Ref.
District conditional on net use (Use a net = Yes)						
District (IRS)	0.62 (0.23–1.66)	0.340	**0.06 (0.01–0.48)**	**0.007**	0.62 (0.25–1.51)	0.291
District (non-IRS)	1.00	Ref.	1.00	Ref.	1.00	Ref.
District conditional on net use (Use a net = No)						
District (IRS)	1.25 (0.51–3.07)	0.626	2.27 (0.79–6.52)	0.128	**0.29 (0.14–0.61)**	**0.001**
District (non-IRS)	1.00	Ref.	1.00	Ref.	1.00	Ref.

During the post IRS-2 survey, clinical malaria was associated with age with higher odds among children <5 years of age (OR = 4.19, 95% CI = 2.59–6.78, p<0.001) and children aged 5 to 14 years (OR = 5.97, 95% CI = 3.55–10.0, p<0.001) compared to adults and females were more likely to have clinical malaria than males (OR = 2.01, 95% CI = 1.37–2.94, p<0.001). Residents of permanent (OR = 0.33, 95% CI = 0.16–0.69, p = 0.003) and semi-permanent houses (OR = 0.50, 95% CI = 0.30–0.85, p = 0.010) had lower odds of clinical malaria than those residing in traditional houses. Residence in the IRS district (OR = 0.29, 95% CI = 0.14–0.61, p = 0.001) and the use of an ITN the previous night (OR = 0.50, 95% CI = 0.29–0.86, p = 0.011) were both associated with lower odds of clinical malaria. However, the interaction between these two variables was not statistically significant (OR = 2.15, 95% CI = 0.73–6.35, p = 0.166).

### Multivariate models of anemia

When vector control was categorized as a 4 level variable, overall rates of anemia at the post IRS-1 survey were 2.0% among those who received IRS alone, 3.0% among those who used an ITN the previous night, 3.1% among those with both ITNs and IRS and 3.2% among those with neither intervention (Fig 3). In a logistic regression model, none of the variables examined was associated with the prevalence of anemia. There was no association between anemia and IRS, ITN use or the combination of these vector control interventions (Table 5).

During the post IRS-2 survey, children <5 years old (OR = 1.81, 95% CI = 1.10–2.97, p = 0.020) had higher odds of anemia than adults while children aged 5 to 14 years (OR = 0.39, 95% CI = 0.18–0.84, p = 0.016) had lower odds of anemia. The prevalence of anemia in the overall population was 1.8% among those who had received IRS alone, 4.2% among those who used an ITN the night before, 2.9% among those with both interventions and 2.6% among those with neither. Again, there was no association between anemia and IRS, ITN use or the combination of IRS and ITNs (Table 5).

Since anemia is more common among children <5 years old, a separate model was run with only this age category. The prevalence of anemia was 1.2% among children who received IRS alone, 1.8% among children with both IRS and ITNs, 9.0% among children who used an ITN the night before but did not receive IRS and 5.4% among children with neither intervention (Fig 3). There were no significant differences among children with the different interventions.

The prevalence of anemia by district and ITN use is shown in Fig 7. In models that included an interaction between ITNs and district as a proxy for IRS, females were more likely to be anemic than males (OR = 2.12, 95% CI = 1.27–3.54, p = 0.004) while children aged 5 to 14 years were less likely to be anemic compared to adults (OR = 0.45, 95% CI = 0.23–0.87, p = 0.017) during the baseline survey. History of ACT use was associated with a higher prevalence of anemia (OR = 3.16, 95% CI = 1.22–8.18, p = 0.018). In the post IRS-1 survey, there was no association between the prevalence of anemia and any of the predictor variables. During the post IRS-2 survey, children <5 years had higher odds of being anemic compared to adults (OR = 1.80, 95% CI = 1.09–2.96, p = 0.021) and children aged 5 to 14 years had lower odds of being anemic compared to adults (OR = 0.39, 95% CI = 0.18–0.84, p = 0.016). Neither the use of ITNs the previous night, district of residence nor the interaction between these variables was significantly associated with anemia during any of the surveys (Table 10). The interaction between district and net use was not statistically significant and there were no significant effects of district or ITN use when conditional on each other (Table 11).

**Table 10:** Multivariate regression model of the impact of different factors on anemia (Hb≤8). The model included an interaction between net use and district (a proxy for IRS). Comparisons that were statistically significant a p<0.05 are indicated in bold.

	Baseline		Post IRS 1		Post IRS 2	
Parameter	OR	*p*	OR	*p*	OR	*p*
Rachuonyo (IRS)	0.89 (0.35, 2.22)	0.796	0.58 (0.28, 1.20)	0.141	0.81 (0.39, 1.67)	0.568
Nyando (non-IRS)	1.00	Ref.	1.00	Ref.	1.00	Ref.
Use a net (Yes)	1.06 (0.58, 1.93)	0.844	0.70 (0.40, 1.22)	0.214	1.22 (0.58, 2.55)	0.597
Use a net (No)	1.00	Ref.	1.00	Ref.	1.00	Ref.
Rachuonyo * Use a net (Yes)	1.59 (0.51, 4.97)	0.422	1.44 (0.62, 3.34)	0.402	0.84 (0.34, 2.06)	0.710
Rachuonyo * Use a net (No)	1.00	Ref.	1.00	Ref.	1.00	Ref.
Nyando * Use a net (Yes)	1.00	Ref.	1.00	Ref.	1.00	Ref.
Nyando * Use a net (No)	1.00	Ref.	1.00	Ref.	1.00	Ref.
Elevation ≥1500	0.41 (0.07, 2.34)	0.313	0.53 (0.18, 1.57)	0.255	0.42 (0.16, 1.11)	0.081
Elevation <1500	1.00	Ref.	1.00	Ref.	1.00	Ref.
Gender (Female)	**2.12 (1.27, 3.54)**	**0.004**	1.26 (0.79, 2.04)	0.333	1.18 (0.80, 1.75)	0.413
Gender (Male)	1.00	Ref.	1.00	Ref.	1.00	Ref.
Age, <5 years	1.45 (0.83, 2.54)	0.189	1.76 (0.90, 3.44)	0.097	**1.80 (1.09, 2.96)**	**0.021**
Age, 5 to 14 years	**0.45 (0.23, 0.87)**	**0.017**	0.55 (0.29, 1.04)	0.068	**0.39 (0.18, 0.84)**	**0.016**
Age, ≥15 years	1.00	Ref.	1.00	Ref.	1.00	Ref.
History of ACT use (Yes)	**3.16 (1.22, 8.18)**	**0.018**	1.81 (0.57, 5.73)	0.312	2.09 (0.96, 4.57)	0.065
History of ACT use (No)	1.00	Ref.	1.00	Ref.	1.00	Ref.
Use coils or sprays (Yes)	1.32 (0.74, 2.35)	0.344	0.58 (0.19, 1.81)	0.351	1.67 (0.87, 3.22)	0.126
Use coils or sprays (No)	1.00	Ref.	1.00	Ref.	1.00	Ref.
House type, Permanent	0.80 (0.36, 1.81)	0.595	0.81 (0.35, 1.88)	0.620	1.06 (0.53, 2.10)	0.872
House type, Semi-permanent	1.29 (0.69, 2.41)	0.432	0.68 (0.35, 1.33)	0.258	0.81 (0.49, 1.36)	0.423
House type, Traditional	1.00	Ref.	1.00	Ref.	1.00	Ref.

**Table 11:** Effect of using a net conditional on district and effect of district conditional on net use on the risk of anemia (Hb<8). Comparisons that were statistically significant a p<0.05 are indicated in bold.

	Baseline		Post IRS 1		Post IRS 2	
Parameter	OR	*p*	OR	*p*	OR	*p*
Net use conditional on district (non-IRS district):						
Use a net (Yes)	1.06 (0.58, 1.93)	0.844	0.70 (0.40, 1.22)	0.214	1.22 (0.58, 2.55)	0.597
Use a net (No)	1.00	Ref.	1.00	Ref.	1.00	Ref.
Net use conditional on district (non-IRS district):						
Use a net (Yes)	1.69 (0.66, 4.33)	0.272	1.01 (0.52, 1.96)	0.978	1.03 (0.65, 1.63)	0.903
Use a net (No)	1.00	Ref.	1.00	Ref.	1.00	Ref.
District conditional on net use (Use a net = Yes)						
District (IRS)	1.41 (0.74–2.68)	0.291	0.83 (0.47–1.47)	0.522	0.68 (0.37–1.28)	0.236
District (non-IRS)	1.00	Ref.	1.00	Ref.	1.00	Ref.
District conditional on net use (Use a net = No)						
District (IRS)	0.89 (0.35–2.22)	0.796	0.58 (0.28–1.20)	0.141	0.81 (0.39–1.67)	0.568
District (non-IRS)	1.00	Ref.	1.00	Ref.	1.00	Ref.

However, in a separate multivariate model including interactions between district and age, children less than 5 years of age living in the IRS district were less likely to be anemic than those living in the non-IRS district during the post IRS-2 survey, after 2 rounds of IRS (OR = 0.28, 95% CI = 0.11–0.72, p = 0.008). The prevalence of anemia among children aged 5 to 14 years (OR = 0.57, 95% CI = 0.12–2.75, p = 0.483) or adults (OR = 1.33, 95% CI = 0.72–2.48, p = 0.358) was not significantly different between the two districts.

## Discussion

In this setting, where neither intervention achieved near universal coverage, the combination of IRS and ITNs in western Kenya compared with ITNs alone was associated with reduced prevalence of malaria infection and clinical malaria, and increased hemoglobin levels and reduced anemia, particularly in children under five years of age. The effects were primarily observed in the third survey which was conducted after 2 rounds of IRS. The reduction in parasite prevalence by over 50% (56% for ITN users and 77% for non-users) was consistent with other studies of IRS in high transmission areas of sub-Saharan Africa [26–28], including a meta-analysis of published studies, which found that IRS reduced parasite prevalence by 62% [29].

These data from western Kenya show that the addition of IRS in this area with high ITN use resulted in a decrease in malaria prevalence when compared with an area with similarly high ITN use but without IRS. The data further suggest that after the implementation of IRS, there may have been limited benefit from the use of ITNs. Models suggested ITNs had a significant impact in the IRS district at baseline and just after the 1^st^ round of IRS. After the 2^nd^ round when the impact of IRS was strongest, there was limited benefit due to ITNs in the IRS district. However, because of biases associated with self-selection of ITN use that cannot be controlled for in our study design, this hypothesis should be tested through a randomized controlled trial. This finding is consistent with surveys from other countries where both ITNs and IRS have been implemented [27, 28] although in many of these surveys, the sample sizes were inadequate to detect differences between users of a single vector control intervention and users of both ITNs and IRS in combination. Analysis of national programs in Ethiopia and Burundi failed to detect a significant added benefit of ITNs and IRS over either intervention by itself [14, 15]. Entomological and modeling studies also suggest that there may be only modest added benefits to combining IRS and ITNs [30–32]. Other modeling studies suggest some added benefit to combining these vector control tools and that combining ITNs with IRS using DDT can lead to elimination [33]. Similarly, observational studies in Bioko Island, mainland Equatorial Guinea and Mozambique showed evidence of an added benefit of both ITNs and IRS [12]. An analysis of nationally representative surveys also showed that the combination of ITNs and IRS reduced the risk of infection compared to ITNs or IRS alone in medium and high transmission areas [13]. Furthermore, a cohort study conducted in the same districts as the current study indicated that IRS plus ITNs reduced the incidence of new infections by 62% compared to ITNs alone [21]. The differences between that study and the current study may be related to the outcome measures with incidence a more sensitive indicator than prevalence. Participants were not cleared of pre-patent parasitemia in these surveys, and participants may have had persistent asymptomatic prevalent malaria to the time of the third survey without having sought treatment. Conversely, a study in Kwa-Zulu Natal demonstrated a reduction in malaria incidence among users of ITNs in an area that had been sprayed suggesting added benefits of ITNs on top of IRS [10]. Given the conflicting results from observational studies, cluster-randomized, controlled studies may provide more definitive data on the combined impact of IRS and ITNs. However, these studies would need to be implemented at scale to capture the community effects of both ITNs and IRS and would need to be conducted in areas with low ITN coverage, which are becoming rare. To date, only three such studies have been conducted. In western Tanzania, there was a clear benefit of adding IRS to ITNs with reduced malaria prevalence among those who received both [16, 17] while in Benin, the addition of either IRS with bendiocarb or bendiocarb-treated plastic sheeting attached to walls combined with targeted or universal ITN coverage in areas of high transmission did not provide a significant benefit in terms of reducing malaria morbidity, infection or transmission [18]. A third trial in the Gambia found no evidence that IRS with DDT provided added protection above high coverage of ITNs [19].

The effects of ITNs and IRS whether used alone or in combination is likely influenced by the behavior and resistance status of the primary malaria vectors. ITNs would be most effective against endophagic vectors that feed primarily late at night while IRS would be most effective against primarily endophilic vectors that spend a significant portion of their adult lives resting inside houses. If a combined effect of ITNs and IRS were to exist, it would be expected to be seen in areas where the predominant mosquito vectors were both strongly endophagic and endophilic. Along the Lake Victoria basin, the primary vector has shifted from *Anopheles gambiae* s.s. and *Anopheles funestus* to predominantly *Anopheles arabiensis* [34]. Data from Nyando and Rachuonyo confirmed that nearly all mosquitoes collected were *An*. *arabiensis* (N. Bayoh, unpublished data). *Anopheles arabiensis* in western Kenya tends to feed predominantly on cattle although a significant portion of its feeds occur on humans[34, 35] and it frequently rests indoors after feeding outdoors but likely exploits outdoor resting sites as well. Given the flexible behavior of *An*. *arabiensis*, it is possible that the implementation of either IRS or ITNs alone provides good protection by driving most mosquitoes out of the house and that the addition of the other adds little protection against malaria infection. Data from this study suggest that IRS is effective against *An*. *arabiensis* despite feeding and resting behaviors that may reduce contact with the insecticide. In areas with more strongly endophagic and endophilic vectors such as *An*. *gambiae* s.s. or *An*. *funestus*, the combined impact of ITNs and IRS may be more prominent.

The predominant malaria vector in the two districts was *An*. *arabiensis* which was observed to be susceptible to pyrethroid insecticides at the time of the study. However, reports from western Kenya since the last survey suggest the resistance is increasing in all the vectors in western Kenya [36–38]. It is possible that low levels of resistance to pyrethroids were present at the time of the IRS but not at sufficient levels to reduce the impact of the IRS. This would suggest that WHO criteria for determining resistance may be slightly conservative but this approach may be appropriate as it would encourage for malaria control programs to switch insecticides before control failure and before resistance becomes too firmly entrenched. However, in the future, countries should follow the recommendations of WHO to avoid IRS with pyrethroids in areas with high coverage of ITNs to avoid intensive selective pressure on vector populations.

When ITNs and IRS were combined into a single variable with either ITNs alone, IRS alone, both or neither as categories, the prevalence of infection and clinical malaria were similar among those using ITNs alone and those who received IRS alone. These findings are similar to those of previous studies which found either no difference in malaria related outcomes among recipients of IRS or users of ITNs, or an advantage to users of ITNs. Cluster- randomized trials conducted in a variety of settings have shown no differences in entomological indicators [39] or malaria incidence [11] among IRS recipients vs ITN users, while one trial in India found that ITNs were more effective in reducing malaria incidence compared with IRS [9]. In programmatic settings, ITNs have also been found to be equal to or better than IRS in several settings. In China, deltamethrin treated nets were equally effective in reducing malaria cases compared to IRS with DDT [40] while in the Solomon Islands the use of permethrin treated nets significantly reduced malaria but could not entirely replace DDT spraying [41]. In other surveys following IRS campaigns in Africa, the impact of ITNs was negligible compared to that of IRS [26–28]. However, in a meta-analysis of nationally representative data from 17 countries, there were few clear differences in the effect of ITNs or IRS. The exceptions were in low transmission or rural settings where IRS was more effective in reducing parasite prevalence [13]. In the current study, the one notable difference between ITNs and IRS was in the prevalence of anemia which was significantly reduced after two rounds of IRS. No effect of ITNs was observed on the prevalence of anemia. Given the widely varying settings with differences in vector behaviors, insecticides used for IRS, the consistency of net use and the quality of both IRS and ITNs, it is not possible to parse out the differences in effectiveness of these two interventions and current recommendations that populations be covered by at least one should be maintained.

The effects of ITNs and IRS are likely affected by both coverage and quality of each intervention. Previous studies have shown that community-wide benefits likely emerge when ITN coverage exceeds 50% and the benefits of bednets may be underestimated when comparing ITN users with non-users who live in communities with high ITN coverage [42]. Given that ITN use was over 50% in all three surveys described here, the impact of ITNs may have been underestimated in this study as non-users may have benefited from a community effect. Similarly, IRS coverage targets are generally over 80% to ensure high community effects as IRS is not thought to confer personal protection [43]. In both rounds of spraying, IRS coverage fell short of this target; however we cannot discount that a community benefit may have occurred none the less, and our comparison of the effectiveness of sprayed versus unsprayed houses on improving malaria indicators similarly may have been underestimated. The impact of ITNs may also have been affected by the quality of the nets. Although we did not estimate the damage to the nets, surveys in Equatorial Guinea have shown that nets in increasingly worse condition were less effective in protecting against malaria infection [43]. In our analyses, we considered all nets to be ITNs whether they were classified as treated or untreated nets as we found no differences in malaria outcomes among users of either ITNs or untreated nets. However, it is possible that the inclusion of nets with increasing numbers of holes and/or declining insecticide content may have increased the prevalence of malaria related outcomes among net users.

The positive effects of IRS were not definitively observed until after the second round of spraying. Previous studies have suggested a cumulative effect of multiple rounds of IRS. In Uganda, malaria prevalence was lower in a district that had received 6 rounds of spraying compared to one that had received only 3 rounds, and malaria prevalence in the district that received 3 rounds was significantly lower than an unsprayed district [28]. Similarly, in a meta-analysis of 13 published studies, IRS effectiveness increased with multiple rounds of spraying [29]. On a longer time scale, Mabaso *et al*. found dramatic declines in parasite rates in countries in southern Africa that continued over the course of multiple years of IRS, in some cases, decades [3]. The lack of an immediate impact in the current study may have been the result of several factors. First, IRS coverage was relatively low during the first round (66.1%) which may have allowed for continued survival of vectors and transmission of malaria. Second, the timing of the post IRS1 survey may have affected the results; the survey was conducted just 3 months after the first IRS campaign was completed and this may not have allowed sufficient time for the clearance of asymptomatic parasitemias. Furthermore, the IRS survey occurred during the low transmission period and the impact of IRS may have been minimal as there was less transmission to prevent at this time.

Our study had several limitations and the results should be interpreted with these caveats. The main limitation to our study design was the non-randomized comparison of two districts. Our baseline data indicate that malaria specific measures were similar before IRS but there were also differences in elevation, house type and net use that may be associated with differences in malaria specific outcomes in subsequent surveys. We applied statistical methods to match residents of the two districts to reduce the degree of heterogeneity in the different populations. However, we cannot rule out pre-existing conditions that may have contributed to reduced malaria in the IRS district. The study was comprised of serial cross-sectional surveys, which complicates the interpretation of the results because of the seasonality of malaria transmission and the chronic nature of malaria infection and anemia. The pattern of malaria infection across the surveys was likely due in part to the timing of each survey. The baseline survey occurred early in the long-rainy season while the second survey was scheduled three months after the completion of IRS; thus, the second survey was done during the cool dry period when transmission is low. The third survey was conducted during a high transmission period after the rainy season.

We found that IRS provided added benefit to ITNs in this area with where ITN use ranged between 30–40% and use of any net ranged between 55% and 65%. However, we did not find evidence that adding ITNs to IRS in western Kenya would provide added benefit, even when IRS coverage was lower than the target 85%. None the less, there may remain settings where adding ITNs to an area where IRS is occurring may be warranted. Non-pyrethroid insecticides for IRS in combination with pyrethroid treated nets may provide added benefit in areas with low levels of pyrethroid resistance in the primary vectors, and the addition of the non-pyrethroid insecticide may slow the spread of pyrethroid resistance, prolonging the effective life of ITNs. At the time of the surveys, there was little pyrethroid resistance in *An*. *arabiensis* in western Kenya [37] although it has been observed in some areas [36] and has increased in western Kenya since the time of these surveys [38]. ITNs may extend the benefits of IRS in areas where the spray application does not extend throughout the full transmission season and during times when IRS is delayed or interrupted.

## Conclusions

These data provide additional evidence that administering IRS in an area with moderate ITN coverage results in reduced malaria prevalence in a moderately high transmission setting of sub-Saharan Africa. In contrast, the added benefit of adding ITNs to IRS is less clear. Both ITNs and IRS are useful tools and have a role in the prevention of malaria. However, given the declining resources for malaria prevention and the limited added benefit of ITNs in areas with IRS, the implementation of these vector control tools should be more strategic to maximize the use of scarce resources. Given its high cost, IRS may be of maximum benefit in areas where elimination may be feasible in the short term, in areas where transmission remains high despite high coverage of ITNs and other interventions, or in areas with high levels of pyrethroid resistance. Future work should focus on developing a strategic approach to implementing ITNs and IRS in a resource constrained environment and the information required (e.g. malaria incidence/burden, insecticide resistance profiles, value for money of each malaria control intervention) to maximize the benefit of available tools.
